# A network-based approach to uncover microRNA-mediated disease comorbidities and potential pathobiological implications

**DOI:** 10.1038/s41540-019-0115-2

**Published:** 2019-11-13

**Authors:** Shuting Jin, Xiangxiang Zeng, Jiansong Fang, Jiawei Lin, Stephen Y. Chan, Serpil C. Erzurum, Feixiong Cheng

**Affiliations:** 1 Department of Computer Science, Xiamen University, Xiamen, 361005 China; 2 School of Information Science and Engineering, Hunan University, Changsha, 410082 China; 3 Genomic Medicine Institute, Lerner Research Institute, Cleveland Clinic, Cleveland, OH 44195 USA; 4 Pittsburgh Heart, Lung, Blood, and Vascular Medicine Institute, Division of Cardiology, Department of Medicine, University of Pittsburgh Medical Center (UPMC) and University of Pittsburgh School of Medicine, Pittsburgh, PA 15213 USA; 5 Department of Pathobiology, Lerner Research Institute, Cleveland Clinic, Cleveland, OH 44195 USA; 6 Respiratory Institute, Cleveland Clinic, Cleveland, OH 44195 USA; 7 Department of Molecular Medicine, Cleveland Clinic Lerner College of Medicine, Case Western Reserve University, Cleveland, OH 44195 USA; 8 Case Comprehensive Cancer Center, Case Western Reserve University School of Medicine, Cleveland, OH 44106 USA

**Keywords:** Computational biology and bioinformatics, Cardiology, Regulatory networks

## Abstract

Disease–disease relationships (e.g., disease comorbidities) play crucial roles in pathobiological manifestations of diseases and personalized approaches to managing those conditions. In this study, we develop a network-based methodology, termed meta-path-based Disease Network (mpDisNet) capturing algorithm, to infer disease–disease relationships by assembling four biological networks: disease–miRNA, miRNA–gene, disease–gene, and the human protein–protein interactome. mpDisNet is a meta-path-based random walk to reconstruct the heterogeneous neighbors of a given node. mpDisNet uses a heterogeneous skip-gram model to solve the network representation of the nodes. We find that mpDisNet reveals high performance in inferring clinically reported disease–disease relationships, outperforming that of traditional gene/miRNA-overlap approaches. In addition, mpDisNet identifies network-based comorbidities for pulmonary diseases driven by underlying miRNA-mediated pathobiological pathways (i.e., hsa-let-7a- or hsa-let-7b-mediated airway epithelial apoptosis and pro-inflammatory cytokine pathways) as derived from the human interactome network analysis. The mpDisNet offers a powerful tool for network-based identification of disease–disease relationships with miRNA-mediated pathobiological pathways.

## Disease comorbidity analysis from microRNA regulatory networks

Identification of disease–disease relationships play essential roles in development of personalized approaches to managing those conditions. A team led by Dr. Feixiong Cheng at Cleveland Clinic developed a network-based methodology (termed mpDisNet) to infer clinically relevant disease–disease relationships from miRNA regulatory networks. mpDisNet shows high performance in identification of clinically reported disease–disease relationships by a unique integration of miRNA–gene–disease networks under the human interactome model. Importantly, mpDisNet successfully identifies a network-based relationship between asthma and chronic obstructive pulmonary disease driven by underlying miRNA-mediated pathobiological pathways. From a translational perspective, if broadly applied, mpDisNet would offer a powerful network-based tool for understanding of clinical comorbidities for multiple complex diseases from heterogeneous biological networks, a significant challenge of precision medicine.

## Introduction

The manifestation and clinical severity of human disease are affected by myriad factors, including genetic, epigenetic, lifestyle, and various environmental variables.^1^ Identification of disease–disease relationships not only offers insights into disease heterogeneity, but also reveal etiology and pathogenesis of disease comorbidities,^2,3^ thus driving development of effective therapeutic strategies.^4,5^ Previous studies designed to map comprehensive disease–disease connections focused mainly on known associations among diseases and associated genes/proteins. However, the predisposition to human disease is dictated by a complex, polygenic, and pleiotropic genetic architecture.^6^ Some complex diseases that are mainly driven by environmental or acquired triggers often display more limited genetic risk. Thus, traditional bioinformatics analysis of genetic risk factors offers limited power to detect the true breadth of complex disease–disease relationships.

Beyond genetic analysis, shared patterns of gene expression have raised possibilities to inspect disease–disease relationships.^6^ Alteration and dysregulation of gene expressions are caused by several biological mechanisms, including microRNA (miRNA) dysregulation. In 1993, Ambros et al. discovered the first type of miRNA (lin-4) in a nematode, revealing for the first time the essential function of miRNA in the posttranscriptional regulation of gene expression.^7^ MiRNAs belong to a class of endogenous, small, non-coding RNAs (~22 nucleotides) and play crucial roles in inhibiting the expression of target mRNAs at the posttranscriptional level.^8^ Specifically, miRNAs regulate target genes by partially or completely pairing with their 3′ UTR region, thereby reducing the stability of the target miRNA or inhibiting translation to downregulate the expression of genes of interest.^9^ This complex regulatory network not only regulates the expression of multiple genes through one miRNA, but also finely regulates the expression of multiple genes by the combination of several miRNAs. Thus, the shared patterns of gene expression regulated by miRNAs may offer possibilities to inspect disease–disease relationships.

Currently, more than 30,000 miRNAs within ~200 species have been identified.^10^ Cumulative empirical evidences show that miRNAs are closely related to the development, progression, and prognosis of multiple diseases, such as pulmonary vascular disease.^11,12^ However, it is not obvious whether ascertaining the comprehensive breadth of miRNA-mediated gene networks offer discerning power to reveal important disease–disease relationships. Recent human protein–protein interactome network modeling shows that network-based approaches have raised possibilities to identify disease–disease relationships^2^ and drug–disease associations.^4^

**Fig. 1:**
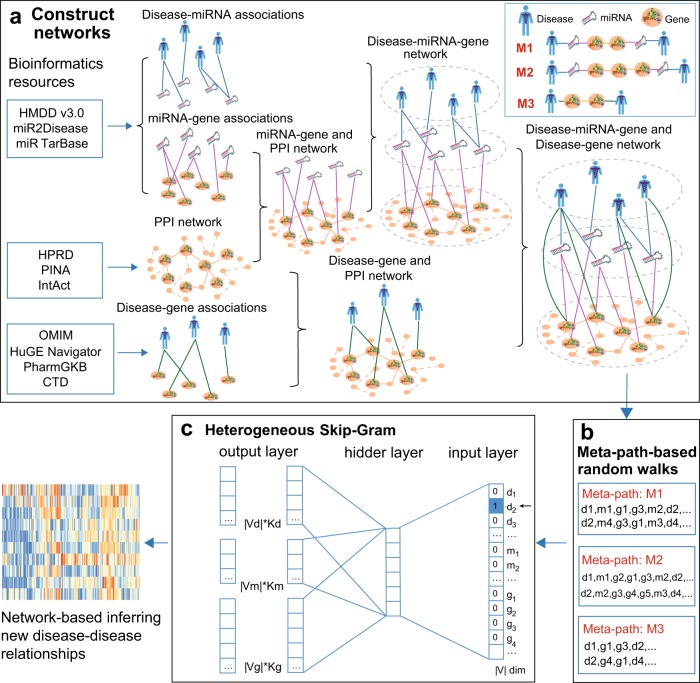
A diagram illustrating mpDisNet. **a** A heterogeneous network is reconstructed by assembling four experimentally validated networks: disease–miRNA, miRNA–gene, disease–gene, and human protein–protein interactome. **b**, **c** MpDisNet, a meta-path based random walk (**b**) to reconstruct the heterogeneous neighbors of a node, uses a heterogeneous skip-gram model (**c**) to solve the network representation of the nodes (see Methods). Herein, three meta-paths are illustrated and used in inferring disease–disease relationships: M1: disease–miRNA–gene–gene–miRNA–disease, M2: disease–miRNA–gene–gene–gene–miRNA–disease, and M3: disease–gene–gene–disease

In this study, we developed a network-based methodology, termed meta-path-based Disease Network (mpDisNet) capturing algorithm, to infer new disease–disease relationships from miRNA-mediated network perspectives. We built a heterogeneous miRNA–gene–disease network by assembling four biological networks: disease–miRNA, miRNA–gene, gene–disease, and the human protein–protein interactome (Table 1). Specifically, mpDisNet searches a specific meta-path (a meta-path is a path linking two specified nodes in a network mode) based on a Random Walk algorithm^13^ to reconstruct the heterogeneous neighbors of a node. Specifically, we utilized a heterogeneous skip-gram model^14^ to solve the network representation of the nodes in mpDisNet (Fig. 1). We found that mpDisNet displayed a higher performance in inferring disease–disease relationships compared with traditional miRNA-overlapping approaches. Via t-distributed stochastic neighbor embedding (t-SNE) analysis,^15^ the reduced dimension graphs generated by the disease–miRNA–gene and disease–gene networks reveal that mpDisNet can effectively distinguish different class of human diseases, offering potential pathobiological implications. We further identified pulmonary disease comorbidities (e.g., lung cancer-asthma and asthma-chronic obstructive pulmonary disease) with potential miRNA-mediated pathobiological mechanisms. If broadly applied, mpDisNet would offer a powerful network-based tool for identification of disease–disease relationships for multiple complex diseases from heterogeneous biological networks.

**Table 1:** A summary of four networks used in this study

Networks	# of nodes		# of links (edges)
Disease–miRNA	diseases	394	7669
	miRNA	691	
miRNA–gene	miRNA	568	163,090
	genes	14,762	
Disease-genes	diseases	394	50,589
	genes	2684	
The human interactome	proteins	16,706	246,995

Note: The number of nodes and edges, and the according data resources are illustrated. More details about those data resources are provided in the Supplementary Methods

## Results

### Pipeline of mpDisNet

MpDisNet infers miRNA-mediated disease–disease relationships based on the topology of multiple networks among diseases, miRNAs, and genes (Fig. 1). The pipeline of mpDisNet has four key steps (see Methods section): (i) network data integration: we reconstructed a heterogenous network by assembling four experimentally validated networks, including disease–miRNA, miRNA–gene, disease–gene, and the human interactome networks (Table 1); (ii) meta-path-based Random Walks: we reconstructed heterogeneous neighbors of the nodes using the random walk of the meta-path and generated instance sequences;^14^ (iii) heterogeneous skip-gram: we generated the multidimensional vector for each disease by the skip-gram from the instance sequences; and (iv) network-based inferring disease–disease relationships: we calculated the disease–disease cosine similarities based on the multidimensional vectors generated from the skip-gram (iii). The detailed pipeline of mpDisNet is illustrated in Fig. 1.

### Performance of mpDisNet

We compared mpDisNet with miRNA-overlap measure on the experimentally validated disease–miRNA association network (see Methods section). Herein, mpDisNet is the result of selecting the meta-path M1 (disease–miRNA–gene–gene–miRNA–disease) and M3 (disease–gene–gene–disease) in an integrated heterogeneous network (Fig. 1). For miRNA-overlap measure, we assume that the set of miRNAs corresponding to disease *A* is *A*_m_, and the corresponding set of disease *B* is *B*_m_. We calculated disease–disease similarity based on overlap measure as below:

1 $$S_{{\mathrm{overlap}}} = \frac{{A_{\mathrm{m}} \bigcap B_{\mathrm{m}}}}{{A_{\mathrm{m}} \bigcup B_{\mathrm{m}}}}$$

**Fig. 2:**
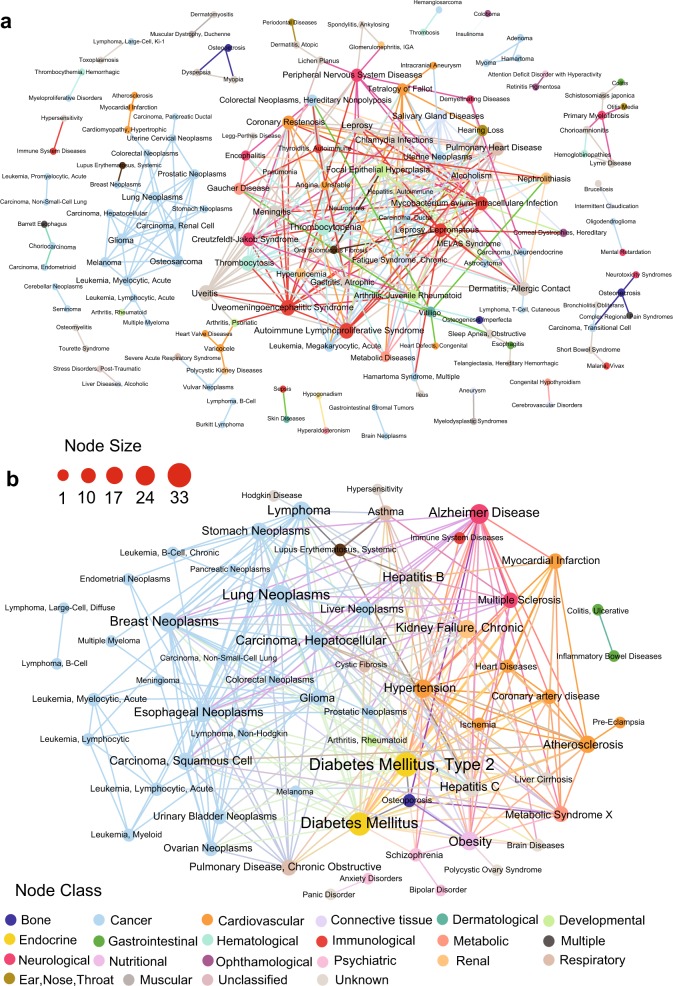
MiRNA-mediated disease–disease networks. Two network graphs of the top 300 disease–disease pairs (Supplementary Table 1) identified by mpDisNet and miRNA-overlap measure, respectively, are shown. **a** A disease–disease network derived from the miRNA-overlap measure. The edges of disease–disease pairs in (**a**) represent the similarity by the miRNA-overlap measure (Eq. 1) alone. The top 300 inferred disease–disease pairs connecting 146 diseases are illustrated. **b** A disease–disease network identified by mpDisNet. The edges of disease–disease pairs in (**b**) represent the similarity from mpDisNet. In this graph, mpDisNet predicts disease-disease relationships by the combined M1 (disease–miRNA–gene–gene–miRNA–disease) and M3 (disease–gene–gene–disease) meta-paths (see Fig. 1). Top 300 inferred disease–disease pairs connecting 61 diseases are illustrated. The node size denotes the degree. The color of nodes is encoded based on the pathobiological categories of diseases. This image is generated by Gephi (https://gephi.org)

We selected the top 300 pairs of the highest similarity disease pairs (Supplementary Table 1) obtained by miRNA-overlap measure and mpDisNet, and plotted two network graphs of miRNA-overlap measure (Fig. 2a) and mpDisNet (Fig. 2b), respectively. The node color of each disease is classified according to the disease pathobiological classification from a previous study.^16^ Overall, the mpDisNet (Fig. 2b) can capture clinically reported disease–disease comorbidities in the same pathobiological categories of specific diseases, outperforming miRNA-overlap measure (Fig. 2a). For example, associations among obesity (Mesh ID: D009765), diabetes mellitus (Mesh ID: D003920), cystic fibrosis (Mesh ID: D003550), osteoporosis (Mesh ID: D010024), and metabolic syndrome X (Mesh ID: D024821) are well captured by mpDisNet (Fig. 2b). For cardiovascular disease, the significant associations among heart disease (myocardial infarction), coronary artery disease, atherosclerosis, ischemia, and hypertension are successfully identified by mpDisNet as well (Fig. 2b). For neurological diseases, the mpDisNet-predicted relationships among schizophrenia, bipolar disorder, and Alzheimer’s disease were consistent with a recent study.^6^ Finally, multiple types of cancer are found to share a strong association identified by mpDisNet, consistent with recent pan-cancer studies.^17,18^ Altogether, mpDisNet identifies potentially well-known disease–disease relationships.

**Fig. 3:**
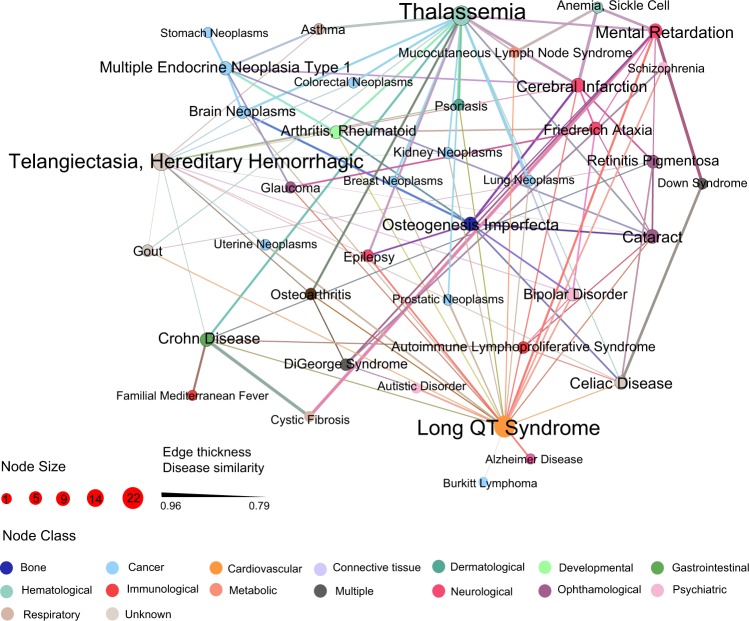
A discovered miRNA-mediated disease–disease network by mpDisNet. In this network, 100 clinically reported disease–disease pairs connecting 39 diseases identified by mpDisNet, while they cannot be identified by miRNA-overlap measure, are shown. The node size denotes the degree. The color of nodes is encoded based on the pathobiological categories of diseases. The weight of edges (disease–disease pairs) denote the predicted score by mpDisNet. This image is generated by Gephi (https://gephi.org)

To validate performance of mpDisNet further, we collected 220 clinically reported disease–disease pairs from a previous study.^19^ We found that these 220 disease–disease pairs can be correctly re-identified by mpDisNet. However, miRNA-overlap measure can only identify 120 pairs. We plotted the network map (Fig. 3) of mpDisNet-predicted 100 comorbid disease pairs (Supplementary Table 2) which are not identified by miRNA-overlap measure. For example, mpDisNet successfully identifies the associations of autoimmune lymphoproliferative syndrome with bipolar disorder, cataract, celiac disease, and Crohn disease. In addition, cerebral infarction is associated with several diseases or syndromes, including friedreich ataxia, long QT Syndrome, multiple endocrine neoplasia Type 1, osteogenesis imperfecta, retinitis pigmentosa, telangiectasia, hereditary hemorrhagic, and thalassemia, identified by mpDisNet as well (Fig. 3 and Supplementary Table 2).

**Fig. 4:**
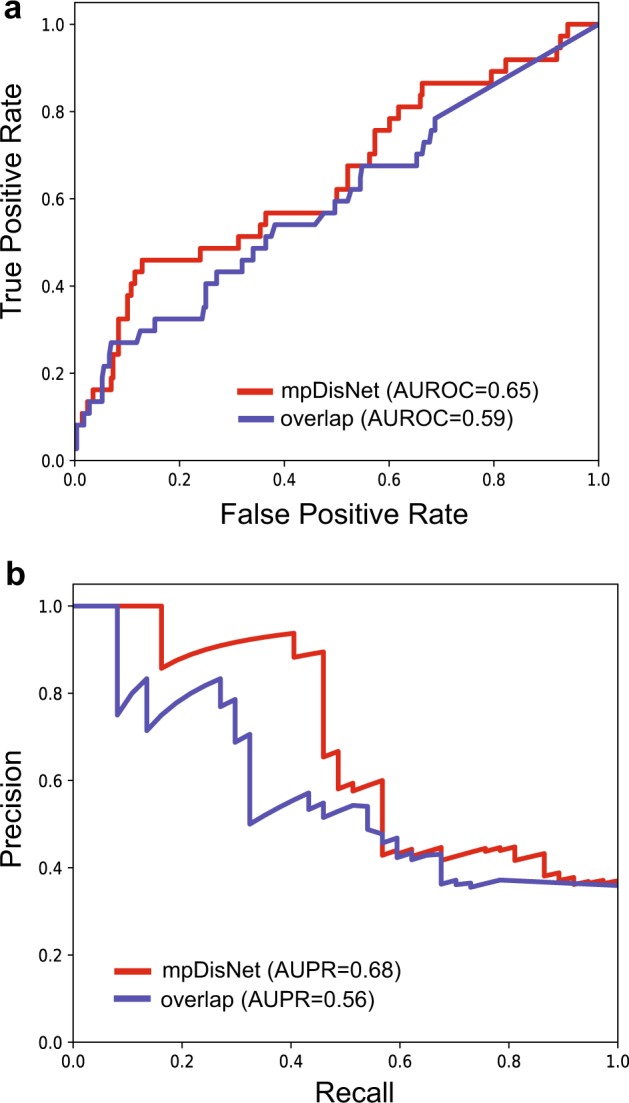
Performance comparison between mpDisNet and miRNA-overlap measure. The receiver operating characteristic (ROC) and precision-recall (PR) curves are plotted relying on the 66 clinically reported disease–disease pairs as the external validation set (Supplementary Table 3). The red curve is generated by mpDisNet and the gray curve by the miRNA-overlap measure (simple measure). The area under ROC (AUROC) and PR curves (AUPR) are provided

We next turned to evaluate the receiver operating characteristic (ROC) and precision-recall curves based on 66 clinically reported disease–disease pairs (Supplementary Table 3) derived from the previously published implicit semantic similarity measure.^20^ We found that mpDisNet showed a reasonable accuracy (the area under ROC [AUROC = 0.65] and the area under precision-recall curve [AUPR] = 0.68, Fig. 4) in inferring the clinically reported disease–disease pairs, outperforming that of miRNA-overlap measure (AUROC = 0.59 and AUPR = 0.56, Fig. 4). In addition, mpDisNet showed a reasonable accuracy (AUROC = 0.67 and AUPR = 0.66) in inferring the clinically reported disease–disease pairs on an external validation set,^21^ revealing high generalizability. Altogether, mpDisNet reveals high accuracy in inferring disease–disease relationships, outperforming traditional miRNA-overlap measure.

### Biological interpretation of mpDisNet

**Fig. 5:**
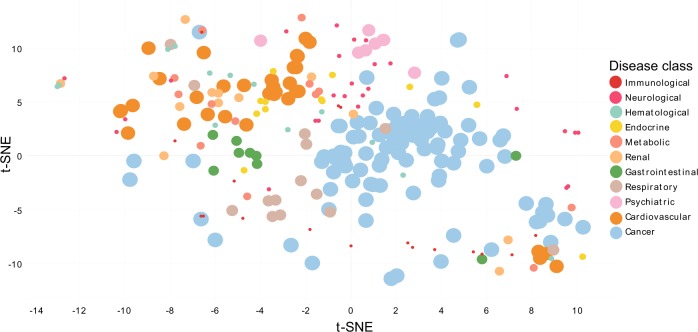
The dimensional reduction visualizes the latent vectors learned by mpDisNet. The latent vectors learned by mpDisNet by combining M1 (disease–miRNA–gene–gene–miRNA–disease) and M3 (disease–gene–gene–disease) meta-paths on an integrated network of disease–gene and disease–miRNA–gene (Fig. 1). We only illustrated the diseases with the well-defined pathobiological category with at least seven types of diseases. The diseases are classified according to the clinically annotated pathobiological classification data (color key) from a previous study.^16^

We next turned to investigate whether the underlying miRNA-mediated subnetworks identified by mpDisNet can offer potential pathobiological mechanisms for the inferred disease–disease relationships. Specifically, we integrated two networks into a single heterogeneous network and evaluated two meta-paths M1 (disease–miRNA–gene–gene–miRNA–disease) and M3 (disease–gene–gene–disease) as shown in Fig. 1. The multidimensional vectors of the two meta-paths were obtained by random walk and skip-gram, and then the multidimensional vectors were concatenated to infer disease–disease relationships (see Methods). We then performed dimensionality reduction visualization analysis using a t-SNE algorithm.^22^ We removed diseases with unknown classification and kept diseases with well-known pathobiological annotations with at least seven types of diseases in each category. In the dimensionality reduction diagram (Fig. 5), a closer distance between two diseases reveals a higher relevant pathobiological relationship. We found that the same pathobiological categories of diseases are clustered by the multidimensional vectors (Fig. 5), indicating that the underlying miRNA-mediated pathobiological pathways can be identified by mpDisNet.

### Network-based identification of miRNA-mediated pathobiological pathways between lung cancer and asthma

As shown in Fig. 2b, we found a strong association of cancers (e.g., lung neoplasms) with asthma and COPD. This finding is consistent with recent meta-analyses, suggesting the potential associations of COPD and asthma with several cancer types such as lung cancer.^23,24^ For example, shortness of breath and respiratory distress often increase the suffering of advanced-stage lung cancer patients.^23,24^ However, the underlying disease pathways for lung cancer-associated asthma remain unclear. Asthma is a condition characterized by chronic inflammation of the lungs, including airway hyper-reactivity, excessive mucous formation, and respiratory obstruction. We asserted that lung cancer-associated asthma may be caused from tumor cell microenvironments, such as cross-talk pro-inflammatory pathway. For example, recent studies showed that micro-environmental inflammation by tumor cell-immune cell cross-talk may induce lung cancer-associated pulmonary hypertension.^25,26^

**Fig. 6:**
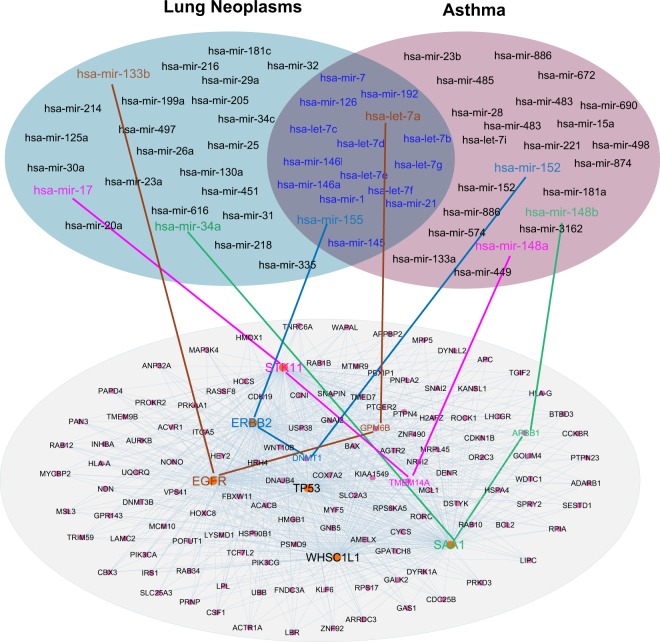
Network-based identification of miRNA-mediated pathobiological pathways for lung cancer-associated asthma. Networks illustrates the relevant miRNA sets between lung cancer and asthma. The overlapping area of two networks denotes the commonly overlapped miRNAs between lung cancer and asthma within the human protein–protein interactome network model. The subnetwork is identified by searching the meta-paths from the human protein–protein interactome network through the random walk of miRNAs between lung cancer and asthma. Four meta-path M1 (disease–miRNA–gene–gene–miRNA–disease) random walks between lung cancer and asthma validated by literature data are highlighted

We therefore performed a multi-layer human interactome network analysis to inspect the miRNA-mediated pathobiological pathways for lung cancer-associated asthma via mpDisNet (Fig. 6). For example, two highlighted miRNAs, hsa-mir-7a and hsa-mir-155, play important roles in both lung cancer^27,28^ and asthma,^29,30^ which are involved in multiple meta-paths in Fig. 6. Hsa-mir-34a was reported as a tumor suppressor gene by inhibiting non-small cell lung cancer (NSCLC) growth and suppressing the CD44hi stem-like NSCLC cells.^31,32^ We found that a meta-path of hsa-mir-34a-SAA1-APBB1 may involve in the lung cancer-associated asthma by meta-path-based network analysis within the human protein–protein interactome (Fig. 6). *SSA1*, encoding serum amyloid A1, activates the NLRP3 inflammasome and promotes asthma in mice.^33^ Thus, hsa-mir-34a that mediates lung tumor growths, may involve in inflammasome-mediated pathways in asthma as well.

We next examined whether we can identify novel miRNA-mediated pathways for lung cancer-associated asthma. Figure 6 reveals that a meta-path of hsa-mir-17-STK11/LKB1 plays a key role in lung cancer by regulating cancer cell metabolism.^34–36^ STK11/LKB1 is a central regulator of T cell development, activation and metabolism.^37^ In addition, the T cell plays an important functional role in asthma as well.^38^ Collectively, hsa-mir-17-STK11/LKB1 may offer a potential pathobiological pathway for lung cancer-associated asthma. In summary, potential miRNA-mediated disease pathways captured by mpDisNet offer candidate biomarkers in understanding of pathobiological mechanisms of lung cancer-associated asthma. However, these candidate network biomarkers identified by mpDisNet are warranted by experimental or clinical validation further.

### Network-based identification of miRNA-mediated pathobiological pathways between COPD and asthma

Asthma and COPD are obstructive pulmonary diseases that have affected millions of people all over the world.^39^ They are two diseases with differences in etiology, symptoms, type of airway inflammation, inflammatory cells, mediators, consequences of inflammation, response to therapy and course.^39^ The similarities in airway inflammation in severe asthma and COPD and good response to combination therapies in both diseases suggest that they may share some pathophysiologic characteristics.^40,41^

**Fig. 7:**
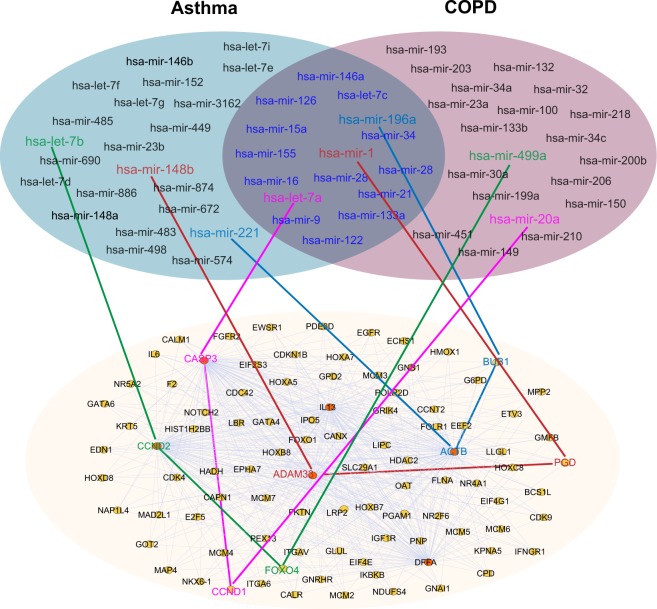
Network-based identification of miRNA-mediated pathobiological pathways between COPD and asthma. Networks illustrate the relevant miRNA sets between COPD and asthma. The overlapping area of the two sets represents the commonly overlapped miRNAs between COPD and asthma within the human protein–protein interactome network model. The subnetwork is identified by searching the meta-paths from the human protein–protein interactome network through the random walk of miRNAs between COPD and asthma. Four meta-path M1 (disease–miRNA–gene–gene–miRNA–disease) random walks between COPD and asthma validated by literature data are highlighted

We next turned to inspect the miRNA-mediated pathways between asthma-COPD. Both hsa-let-7a (differentially expressed in patients with severe asthma^42^) and hsa-let-7b play important roles in asthma by targeting pro-inflammatory pathways.^29^ We found two meta-paths, including hsa-let-7a-CASP3-CCND1-hsa-mir-20a and hsa-let-7b-CCND2-FOXO4-hsa-mir-499a between asthma and COPD, via mpDisNet (Fig. 7). Genetic studies and in vitro observations have shown potential associations of CCND1 and CCND2 with asthma and COPD.^43–45^ In addition, CASP3 was reported to play a functional role in airway epithelial apoptosis^46,47^ and pro-inflammatory cytokines (FOXO4) may contribute to regulation of muscle atrophy and smooth muscle cell migration.^48,49^ Altogether, miRNA-mediated airway epithelial apoptosis and pro-inflammatory cytokine pathways (hsa-let-7a and hsa-let-7b) may offer potential mechanisms for the overlapping syndrome between asthma and COPD. In addition, several mpDisNet-predicted meta-paths, such as hsa-mir-148b-ADAM33-PGD-hsa-mir-1 and hsa-mir-221-ACTB-BUB1-hsa-mir-196a (Fig. 7) may offer new pathobiological pathways to explain the asthma-COPD comorbidity as well.^50–54^

## Discussion

Understanding of disease–disease relationships is important for the diagnosis, prevention, and treatment of the human disease. Most of the existing comorbid data are from the medical records analysis of clinical patients.^3^ This method requires a large amount of data calculation and has many interference factors. Recent remarkable development of systems biology technologies and network medicine approaches raised possibilities to predict disease comorbidities from human protein–protein interactome.^2,3^ In order to integrate biological networks to predict disease–disease relationships, we presented a network-based methodology, termed mpDisNet, to infer disease–disease relationships from miRNA regulatory network perspective.

Specifically, we constructed a comprehensive, multi-layer biological network connecting diseases, miRNA, and genes. We employed a skip-gram algorithm to obtain the multidimensional feature vectors of disease and then calculated the disease–disease similarities from the reduced informative multidimensional vectors. We demonstrated that mpDisNet can identify both clinically reported and new disease–disease associations, outperforming miRNA-overlap measure. Moreover, mpDisNet offers miRNA-mediated pathobiological pathways by searching miRNA meta-paths from the human protein–protein interactome, as we showcased for lung cancer-associated asthma and asthma-COPD. However, comprehensive validation for more mpDisNet-predicted disease–disease relationships are warranted in the future.

We highlighted several significant contributions in the current study. We assembled four comprehensive networks, including disease–miRNA, miRNA–genes, disease–gene, and the human protein–protein interactome to search the meta-paths by mpDisNet. In this way, we can utilize the complementary information from different biological networks compared with traditional network-based approaches using single type of data.^55,56^ Network analysis further shows that integrating miRNA-mediated network can improve the capability in inferring disease–disease relationships, offering a new network-based tool for assessment of disease comorbidities. In addition, the network-based framework presented in mpDisNet could be applied for prediction of drug–target interactions, gene–gene (protein–protein) interactions, RNA–RNA interactions, and other biological networks as well. Finally, the new disease–disease relationships inferred by mpDisNet may offer potential candidate network biomarkers for better understanding of underlying pathobiological pathways from miRNA network perspective.

We acknowledged several potential limitations in current network-based framework of mpDisNet. First, when the known miRNA associated with disease is fewer, the comorbidity between disease pairs computed by miRNA-mediated networks may be false positive. Second, potential literature data bias (e.g., degree/connectivity of well-studied miRNAs/proteins) may generate a potential false positive rate. Third, each random walk requires a specific meta-path, and the choice of this single meta-path may also affect performance of mpDisNet. In the future, we may improve mpDisNet by integrating more comprehensive biological networks, analyzing the relevant associations in tissue-specific networks in which the disease occurs, adopting more flexible random walk strategies.

In summary, this study offers a network-based, systems biology methodology for comprehensive identification of disease–disease relationships from miRNA regulatory network perspective. From a translational perspective, if broadly applied, mpDisNet would offer a powerful network-based tool for understanding of clinical comorbidities for multiple complex diseases from heterogeneous biological networks, a significant challenge of precision medicine.

## Methods

### Reconstruction of heterogeneous networks

We reconstructed a heterogenous miRNA–gene–disease network by assembling four types of networks: (a) disease–miRNA, (b) miRNA–gene, (c) disease–gene, and (d) the human protein–protein interactome networks.

#### Disease–miRNA network

We collected experimentally validated disease–miRNA associations from two databases: miR2Disease^57^ and HMDD v3.0.^58^ All disease terms were annotated by Medical Subject Headings (MeSH) and Unified Medical Language System (UMLS) vocabularies.^59^ The disease–miRNA associations in two databases were combined and the duplicate associations were removed. Finally, we kept a total of 7669 associations connecting 691 miRNAs with 394 diseases in this study.

#### miRNA–gene network

We collected the known miRNA targets to build miRNA–gene networks from miRTarBase database.^60^ We annotated all protein-coding genes using gene Entrez ID, chromosomal location, and the official gene symbols from the National Center for Biotechnology Information (NCBI) database.^61^ In this study, we only kept the data from Homo sapiens. After excluding duplicate associations, 163,090 miRNA–gene associations connecting 568 miRNAs with 14,762 human genes were used.

#### Disease–gene network

We assembled disease–gene associations from four public databases: the Online Mendelian Inheritance in Man (OMIM),^62^ HuGE Navigator,^63^ PharmGKB,^64^ and Comparative Toxicogenomics Database (CTD).^65^ All disease terms were annotated using MeSH vocabularies,^66^ and the genes were annotated using the Entrez IDs and official gene symbols from the NCBI database.^66^ Duplicated pairs from different data sources were deleted. In total, we obtained 50,589 disease–gene associations connecting 2684 genes with 394 unique disease terms.

#### The human protein–protein interactome

To build a comprehensive human protein–protein interactome, we focused on high-quality protein–protein interactions (PPIs) with five types of experimental evidences: (i) Binary PPIs tested by high-throughput yeast-two-hybrid (Y2H) systems;^67,68^ (ii) Kinase-substrate interactions by literature-derived low-throughput and high-throughput experiments; (iii) Literature-curated PPIs identified by affinity purification followed by mass spectrometry (AP-MS), Y2H and by literature-derived low-throughput experiments; (iv) PPIs from protein three-dimensional (3D) structures; and (v) Signaling networks supported by literature-derived low-throughput experiments. The genes were mapped to their Entrez ID based on the NCBI database^61^ as well as their official gene symbols based on GeneCards (http://www.genecards.org/). Duplicated PPIs and all computationally predicted data, such as evolutionary analysis, metabolic associations, and gene co-expression data, were deleted. The resulting updated human interactome used in this study includes 246,995 PPIs connecting 16,706 unique proteins. The detailed descriptions are provided in our recent studies.^4,5^

### Meta-path-based random walks

We employed a meta-path-based random walk to capture the semantic and structural correlation between different types of nodes. Given a heterogeneous network, G = (*V*, *E*, *F*), and meta-path, $P:V_1\mathop { \to }\limits^{R_1} V_2\mathop { \to }\limits^{R_2} V_3\mathop { \to }\limits^{R_3} \cdots V_f\mathop { \to }\limits^{R_f} V_{f + 1} \cdots \mathop { \to }\limits^{R_{l - 1}} V_l$, the transition probability in step *i* was defined as follows:

2 $$P({v^{i + 1}} \vert v_f^i,p) = \left\{ {\begin{array}{*{20}{c}}{\frac{1}{{\vert{N_{f + 1}}(v_f^i)\vert}}}&{({v^{i + 1}},v_f^i) \in E,\emptyset ({v^{i + 1}}) = f + 1} \\0&{({v^{i + 1}},v_f^i) \in E,\emptyset ({v^{i + 1}})\, \ne \,f + 1} \\0&{({v^{i + 1}},v_f^i) \;\notin\; E}\end{array}} \right.$$

where $v_f^i \in V_f$, and $N_{f + 1}(v_f^i)$ represent the set of nodes belonging to the type, *V*_*f*+1_, in the neighborhood of node, $v_f^i$. In other words, $v^{i + 1} \in V_{f + 1}$, walking is on the condition of a preset meta-path, *P*. Moreover, meta-paths are generally used on symmetric paths, that is, its first node type *V*_1_ is the same with the last one *V*_l_, facilitating its recursive for random walks, i.e.,

3 $$P\left( {v^{i + 1}{\mathrm{|}}v_f^i} \right) = p\left( {v^{i + 1}{\mathrm{|}}v_l^i} \right),\,{\mathrm{if}}\,f = l$$

The meta-path-based random walk strategy ensures that the semantic relationships among different types of nodes are properly conserved in the reconstructed heterogeneous network.

### Heterogeneous skip-gram

Furthermore, we employed a heterogeneous skip-gram representation learning model.^13^ The heterogeneous skip-gram is a modification based on the original Skip-gram model, by adding the superposition of different node types. For a heterogeneous network, G = (*V*, *E*, *F*), each node, *ν*, and each edge, *e*, are associated with their mapping functions, $\varphi \left( v \right):V \to F_V(\left| {F_V} \right|\, > \,1)$ and $\psi \left( e \right):E \to F_E$, respectively. Given a node, *ν*, maximizes the probability that the heterogeneous context, *N*_*f*_(*ν*), $\left( {f \in F_V} \right)$ is as follows:

4 $${\mathrm{argmax}}_{\theta} \mathop {\sum }\limits_{v\epsilon V} \mathop {\sum }\limits_{f\epsilon F_V} \mathop {\sum }\limits_{c_f\epsilon N_f(v)} {\mathrm{log}}\,p\left( {c_f{\mathrm{|}}v;\theta } \right)$$

where *N*_*f*_(*ν*) denotes the neighborhood of *ν* with the *f*th type of nodes. The conditional probability, $p\left( {c_f{\mathrm{|}}v;\theta } \right)$, is defined as a softmax function^69^ and adjusted to a specific node type,^70^ *f*, as follows:

5 $$p\left( {c_f{\mathrm{|}}v;\theta } \right) = \frac{{e^{X_{c_f}\cdot X_v}}}{{\mathop {\sum }\nolimits_{u_f\epsilon V_f} e^{X_{u_f}\cdot X_v}}}$$

where *X*_*v*_ is the *v*th row of *X*, which is the embedding vector for node *v*; *V*_*f*_ represents the node type set of type, *f*, in the network. This specifies a multinomial distribution for each type in the output layer of the last layer of skip-gram. According to the negative sampling^71^ in Word2vec,^72^ the above function is defined as follows:

6 $${O}(X) = \log \sigma \left( {X_{c_f} \cdot X_v} \right) + \mathop {\sum}\limits_{m = 1}^M { \mathrm{E}_{u_f^m\sim P_f\left( {u_f} \right)}} \left[ {{\mathrm{log}}\sigma \left( { - X_{u_f^m} \cdot X_v} \right)} \right]$$

where ${\mathrm{\sigma }}\left( x \right) = \frac{1}{{1 + e^{ - x}}}$ and *P*_*f*_(*u*_*f*_) are pre-defined distributions by the type of node of neighbor, *c*_*f*_, that aims to predict from which a negative node $u_f^m$ is drawn from for *M* times.

The gradients of the above pre-defined distributions are derived as follows:

7 $$\frac{{\partial}O(X)}{{\partial X_{u_f^m}}} = \left( {\sigma \left( {X_{u_f^m} \cdot X_v - {\mathbf{I}}_{c_f}[u_f^m]} \right)} \right)X_v$$

8 $$\frac{{\partial {O}(X)}}{{\partial X_v}} = \mathop {\sum }\limits_{m = 0}^M \left( {\sigma \left( {X_{u_f^m} \cdot X_v - {\mathbf {I}}_{c_f}[u_f^m]} \right)} \right)X_{u_f^m}$$

where ${\mathbf I}_{c_f}[u_f^m]$ is an indicator function to indicate whether $u_f^m$ is the neighborhood context node *c*_*f*_. When *m* = 0, then $u_f^0 = c_f$. The model is optimized by using the stochastic gradient descent algorithm.^73^

### Network-based inferring disease–disease relationships

The network-based similarities between two diseases can be calculated based on single meta-path or multiple meta-paths. In this study, we evaluated three meta-paths (M1, M2, M3) to infer disease–disease relationships. For M1 (disease–miRNA–gene–gene–miRNA–disease) as shown in Fig. 1, we randomly walked in disease–miRNA–gene heterogeneous network based on meta-path M1 for 50 steps. Each walk includes 251 nodes. We run 1000 random walks for each disease and 1000 random walk instance sequences are generated. By inputting all the sequences into heterogeneous skip-gram, we obtained the representation vectors of each disease. Then, we calculated the cosine similarity between diseases based on these vectors. In this way, we calculated the disease similarity for meta-path M2 (disease–miRNA–gene–gene–gene–miRNA–disease), M3 (disease–gene–gene–disease) as well. We predicted disease–disease relationships based on multiple meta-paths by concatenating the representation vectors learned from each meta-path and then calculated the cosine similarity between the concatenated vectors. Therefore, we assembled a disease–miRNA–gene network and a disease–gene network into a heterogeneous network. In this integrated heterogeneous network, we selected the meta-paths M1 and M3, respectively. The multidimensional vectors of the two meta-paths can be obtained by random walk and skip-gram, and then the multidimensional vectors were concatenated to infer disease–disease relationships. The detailed network-based analyses are provided in our recent studies.^4,5,74^

## Supplementary information

Supplementary Tables 1-4

nr-reporting-summary

## Data Availability

The authors declare that the data supporting the findings of this study are available within the paper and its supplementary information files, and https://github.com/ChengF-Lab/mpDisNet.

## Code availability

Custom codes used in this study are available at https://github.com/ChengF-Lab/mpDisNet.
