# Supplementary material for: A network-based approach to uncover microRNA-mediated disease comorbidities and potential pathobiological implications
Source: NPJ Syst Biol Appl. 2019 Nov 13;5:41. doi: 10.1038/s41540-019-0115-2 (PMC6853960; doi:10.1038/s41540-019-0115-2)
Supplement: Supplementary file 1 — Supplementary Tables 1-4 [file 41540_2019_115_MOESM1_ESM.pdf]

**Supplementary Table 1:** The top 300 pairs of the highest similarity disease pairs obtained by miRNA-overlap measure and mpDisNet.

| Source | Target | similarity |
|---|---|---|
| Arthritis, Juvenile Rheumatoid | Alcoholism | 0.5 |
| Autoimmune Lymphoproliferative Syndrome | Alcoholism | 0.5 |
| Carcinoma, Neuroendocrine | Astrocytoma | 0.6666667 |
| Carcinoma, Renal Cell | Carcinoma, Hepatocellular | 0.3502825 |
| Carcinoma, Transitional Cell | Bronchiolitis Obliterans | 0.5 |
| Chlamydia Infections | Alcoholism | 1 |
| Chlamydia Infections | Arthritis, Juvenile Rheumatoid | 0.5 |
| Chlamydia Infections | Autoimmune Lymphoproliferative Syndrome | 0.5 |
| Choriocarcinoma | Barrett Esophagus | 0.4117647 |
| Choriocarcinoma | Carcinoma, Endometrioid | 0.3513514 |
| Colorectal Neoplasms | Cardiomyopathy, Hypertrophic | 0.3482143 |
| Colorectal Neoplasms, Hereditary Nonpolyposis | Angina, Unstable | 0.5 |
| Congenital Hypothyroidism | Cerebrovascular Disorders | 1 |
| Coronary Restenosis | Alcoholism | 1 |
| Coronary Restenosis | Arthritis, Juvenile Rheumatoid | 0.5 |
| Coronary Restenosis | Autoimmune Lymphoproliferative Syndrome | 0.5 |
| Coronary Restenosis | Chlamydia Infections | 1 |
| Creutzfeldt-Jakob Syndrome | Alcoholism | 1 |
| Creutzfeldt-Jakob Syndrome | Arthritis, Juvenile Rheumatoid | 0.5 |
| Creutzfeldt-Jakob Syndrome | Autoimmune Lymphoproliferative Syndrome | 0.5 |
| Creutzfeldt-Jakob Syndrome | Chlamydia Infections | 1 |
| Creutzfeldt-Jakob Syndrome | Coronary Restenosis | 1 |
| Demyelinating Diseases | Carcinoma, Neuroendocrine | 0.5 |
| Dermatitis, Allergic Contact | Carcinoma, Neuroendocrine | 0.6666667 |
| Dermatitis, Allergic Contact | Colorectal Neoplasms, Hereditary Nonpolyposis | 0.5 |
| Dermatitis, Allergic Contact | Demyelinating Diseases | 0.6666667 |
| Encephalitis | Colorectal Neoplasms, Hereditary Nonpolyposis | 0.5 |
| Focal Epithelial Hyperplasia | Astrocytoma | 0.5 |
| Focal Epithelial Hyperplasia | Autoimmune Lymphoproliferative Syndrome | 0.5 |
| Focal Epithelial Hyperplasia | Carcinoma, Ductal | 0.5 |
| Focal Epithelial Hyperplasia | Corneal Dystrophies, Hereditary | 0.5 |
| Focal Epithelial Hyperplasia | Dermatitis, Allergic Contact | 0.5 |
| Gastritis, Atrophic | Angina, Unstable | 0.5 |
| Gastritis, Atrophic | Colorectal Neoplasms, Hereditary Nonpolyposis | 1 |
| Gastritis, Atrophic | Dermatitis, Allergic Contact | 0.5 |
| Gastritis, Atrophic | Encephalitis | 0.5 |
| Gastrointestinal Stromal Tumors | Brain Neoplasms | 0.4347826 |
| Gaucher Disease | Angina, Unstable | 0.5 |
| Gaucher Disease | Colorectal Neoplasms, Hereditary Nonpolyposis | 1 |
| Gaucher Disease | Dermatitis, Allergic Contact | 0.5 |
| Gaucher Disease | Encephalitis | 0.5 |
| Gaucher Disease | Gastritis, Atrophic | 1 |
| Glioma | Carcinoma, Hepatocellular | 0.4227642 |
| Glioma | Carcinoma, Renal Cell | 0.3991228 |
| Glioma | Colorectal Neoplasms | 0.3489583 |
| Hamartoma | Adenoma | 1 |
| Hamartoma Syndrome, Multiple | Focal Epithelial Hyperplasia | 0.5 |
| Hearing Loss | Fatigue Syndrome, Chronic | 1 |
| Hemoglobinopathies | Chorioamnionitis | 0.5 |
| Hepatitis, Autoimmune | Focal Epithelial Hyperplasia | 0.5 |
| Hyperuricemia | Fatigue Syndrome, Chronic | 1 |
| Hyperuricemia | Hearing Loss | 1 |
| Hypogonadism | Hyperaldosteronism | 1 |
| Ileus | Hamartoma Syndrome, Multiple | 0.5 |
| Immune System Diseases | Hypersensitivity | 0.35 |

| | | |
|---|---|---|
| Insulinoma | Coloboma | 1 |
| Intermittent Claudication | Brucellosis | 0.5 |
| Legg-Perthes Disease | Encephalitis | 0.5 |
| Leprosy | Alcoholism | 0.5 |
| Leprosy | Autoimmune Lymphoproliferative Syndrome | 1 |
| Leprosy | Chlamydia Infections | 0.5 |
| Leprosy | Coronary Restenosis | 0.5 |
| Leprosy | Creutzfeldt-Jakob Syndrome | 0.5 |
| Leprosy | Focal Epithelial Hyperplasia | 0.5 |
| Leprosy, Lepromatous | Astrocytoma | 0.5 |
| Leprosy, Lepromatous | Autoimmune Lymphoproliferative Syndrome | 0.5 |
| Leprosy, Lepromatous | Carcinoma, Ductal | 0.5 |
| Leprosy, Lepromatous | Corneal Dystrophies, Hereditary | 0.5 |
| Leprosy, Lepromatous | Dermatitis, Allergic Contact | 0.5 |
| Leprosy, Lepromatous | Focal Epithelial Hyperplasia | 1 |
| Leprosy, Lepromatous | Hamartoma Syndrome, Multiple | 0.5 |
| Leprosy, Lepromatous | Hepatitis, Autoimmune | 0.5 |
| Leprosy, Lepromatous | Leprosy | 0.5 |
| Leukemia, Lymphocytic, Acute | Arthritis, Rheumatoid | 0.375 |
| Leukemia, Megakaryocytic, Acute | Fatigue Syndrome, Chronic | 0.5 |
| Leukemia, Megakaryocytic, Acute | Hearing Loss | 0.5 |
| Leukemia, Megakaryocytic, Acute | Hyperuricemia | 0.5 |
| Leukemia, Myelocytic, Acute | Carcinoma, Renal Cell | 0.40625 |
| Leukemia, Myelocytic, Acute | Glioma | 0.3903509 |
| Leukemia, Myelocytic, Acute | Leukemia, Lymphocytic, Acute | 0.415493 |
| Leukemia, Promyelocytic, Acute | Carcinoma, Non-Small-Cell Lung | 0.40625 |
| Lichen Planus | Dermatitis, Atopic | 0.3636364 |
| Lung Neoplasms | Breast Neoplasms | 0.3404255 |
| Lung Neoplasms | Carcinoma, Renal Cell | 0.4055556 |
| Lung Neoplasms | Colorectal Neoplasms | 0.3884892 |
| Lung Neoplasms | Glioma | 0.3574661 |
| Lung Neoplasms | Leukemia, Myelocytic, Acute | 0.3791209 |
| Lupus Erythematosus, Systemic | Breast Neoplasms | 0.3431373 |
| Lyme Disease | Chorioamnionitis | 1 |
| Lyme Disease | Hemoglobinopathies | 0.5 |
| Lymphoma, B-Cell | Burkitt Lymphoma | 0.3863636 |
| Lymphoma, T-Cell, Cutaneous | Dermatitis, Allergic Contact | 0.4 |
| Melanoma | Carcinoma, Hepatocellular | 0.4540682 |
| Melanoma | Carcinoma, Renal Cell | 0.4380165 |
| Melanoma | Glioma | 0.4522059 |
| Melanoma | Leukemia, Myelocytic, Acute | 0.3895582 |
| Melanoma | Lung Neoplasms | 0.3483607 |
| MELAS Syndrome | Fatigue Syndrome, Chronic | 1 |
| MELAS Syndrome | Hearing Loss | 1 |
| MELAS Syndrome | Hyperuricemia | 1 |
| MELAS Syndrome | Leukemia, Megakaryocytic, Acute | 0.5 |
| Meningitis | Alcoholism | 1 |
| Meningitis | Arthritis, Juvenile Rheumatoid | 0.5 |
| Meningitis | Autoimmune Lymphoproliferative Syndrome | 0.5 |
| Meningitis | Chlamydia Infections | 1 |
| Meningitis | Coronary Restenosis | 1 |
| Meningitis | Creutzfeldt-Jakob Syndrome | 1 |
| Meningitis | Leprosy | 0.5 |
| Metabolic Diseases | Fatigue Syndrome, Chronic | 0.5 |
| Metabolic Diseases | Hearing Loss | 0.5 |
| Metabolic Diseases | Hyperuricemia | 0.5 |

| | | |
|---|---|---|
| Metabolic Diseases | MELAS Syndrome | 0.5 |
| Multiple Myeloma | Leukemia, Lymphocytic, Acute | 0.3510638 |
| Muscular Dystrophy, Duchenne | Dermatomyositis | 0.4482759 |
| Mycobacterium avium-intracellulare Infection | Alcoholism | 1 |
| Mycobacterium avium-intracellulare Infection | Arthritis, Juvenile Rheumatoid | 0.5 |
| Mycobacterium avium-intracellulare Infection | Autoimmune Lymphoproliferative Syndrome | 0.5 |
| Mycobacterium avium-intracellulare Infection | Chlamydia Infections | 1 |
| Mycobacterium avium-intracellulare Infection | Coronary Restenosis | 1 |
| Mycobacterium avium-intracellulare Infection | Creutzfeldt-Jakob Syndrome | 1 |
| Mycobacterium avium-intracellulare Infection | Leprosy | 0.5 |
| Mycobacterium avium-intracellulare Infection | Meningitis | 1 |
| Myelodysplastic Syndromes | Aneurysm | 0.3623188 |
| Myocardial Infarction | Atherosclerosis | 0.3508772 |
| Myocardial Infarction | Cardiomyopathy, Hypertrophic | 0.3627451 |
| Myoma | Adenoma | 1 |
| Myoma | Hamartoma | 1 |
| Myopia | Dyspepsia | 1 |
| Nephrolithiasis | Angina, Unstable | 0.5 |
| Nephrolithiasis | Colorectal Neoplasms, Hereditary Nonpolyposis | 1 |
| Nephrolithiasis | Dermatitis, Allergic Contact | 0.5 |
| Nephrolithiasis | Encephalitis | 0.5 |
| Nephrolithiasis | Gastritis, Atrophic | 1 |
| Nephrolithiasis | Gaucher Disease | 1 |
| Neutropenia | Focal Epithelial Hyperplasia | 0.5 |
| Neutropenia | Leprosy, Lepromatous | 0.5 |
| Oligodendroglioma | Mental Retardation | 1 |
| Oral Submucous Fibrosis | Focal Epithelial Hyperplasia | 0.5 |
| Oral Submucous Fibrosis | Leprosy, Lepromatous | 0.5 |
| Osteonecrosis | Carcinoma, Transitional Cell | 0.5 |
| Osteonecrosis | Complex Regional Pain Syndromes | 0.5 |
| Osteonecrosis | Neurotoxicity Syndromes | 0.5 |
| Osteopetrosis | Dyspepsia | 0.5 |
| Osteopetrosis | Myopia | 0.5 |
| Osteosarcoma | Carcinoma, Hepatocellular | 0.3611111 |
| Osteosarcoma | Carcinoma, Renal Cell | 0.3719807 |
| Osteosarcoma | Colorectal Neoplasms | 0.3575758 |
| Osteosarcoma | Glioma | 0.4329004 |
| Osteosarcoma | Leukemia, Myelocytic, Acute | 0.3492823 |
| Osteosarcoma | Lung Neoplasms | 0.4095745 |
| Osteosarcoma | Melanoma | 0.3846154 |
| Periodontal Diseases | Dermatitis, Atopic | 0.3846154 |
| Peripheral Nervous System Diseases | Autoimmune Lymphoproliferative Syndrome | 0.6666667 |
| Peripheral Nervous System Diseases | Carcinoma, Neuroendocrine | 0.5 |
| Peripheral Nervous System Diseases | Demyelinating Diseases | 0.5 |
| Peripheral Nervous System Diseases | Dermatitis, Allergic Contact | 0.6666667 |
| Peripheral Nervous System Diseases | Glomerulonephritis, IGA | 0.5 |
| Peripheral Nervous System Diseases | Leprosy | 0.6666667 |
| Peripheral Nervous System Diseases | Lichen Planus | 0.375 |
| Pneumonia | Lichen Planus | 0.375 |
| Pneumonia | Peripheral Nervous System Diseases | 0.5 |
| Primary Myelofibrosis | Chorioamnionitis | 0.5 |
| Primary Myelofibrosis | Lyme Disease | 0.5 |
| Primary Myelofibrosis | Otitis Media | 0.5 |
| Prostatic Neoplasms | Carcinoma, Renal Cell | 0.3480392 |
| Prostatic Neoplasms | Glioma | 0.3416667 |
| Prostatic Neoplasms | Leukemia, Myelocytic, Acute | 0.3514851 |

| | | |
|---|---|---|
| Prostatic Neoplasms | Lung Neoplasms | 0.3403141 |
| Prostatic Neoplasms | Osteosarcoma | 0.3537736 |
| Pulmonary Heart Disease | Alcoholism | 1 |
| Pulmonary Heart Disease | Arthritis, Juvenile Rheumatoid | 0.5 |
| Pulmonary Heart Disease | Autoimmune Lymphoproliferative Syndrome | 0.5 |
| Pulmonary Heart Disease | Chlamydia Infections | 1 |
| Pulmonary Heart Disease | Coronary Restenosis | 1 |
| Pulmonary Heart Disease | Creutzfeldt-Jakob Syndrome | 1 |
| Pulmonary Heart Disease | Leprosy | 0.5 |
| Pulmonary Heart Disease | Meningitis | 1 |
| Pulmonary Heart Disease | Mycobacterium avium-intracellulare Infection | 1 |
| Retinitis Pigmentosa | Attention Deficit Disorder with Hyperactivity | 0.375 |
| Salivary Gland Diseases | Astrocytoma | 0.5 |
| Salivary Gland Diseases | Autoimmune Lymphoproliferative Syndrome | 0.5 |
| Salivary Gland Diseases | Carcinoma, Ductal | 0.5 |
| Salivary Gland Diseases | Corneal Dystrophies, Hereditary | 0.5 |
| Salivary Gland Diseases | Dermatitis, Allergic Contact | 0.5 |
| Salivary Gland Diseases | Focal Epithelial Hyperplasia | 1 |
| Salivary Gland Diseases | Hamartoma Syndrome, Multiple | 0.5 |
| Salivary Gland Diseases | Hepatitis, Autoimmune | 0.5 |
| Salivary Gland Diseases | Leprosy | 0.5 |
| Salivary Gland Diseases | Leprosy, Lepromatous | 1 |
| Salivary Gland Diseases | Neutropenia | 0.5 |
| Salivary Gland Diseases | Oral Submucous Fibrosis | 0.5 |
| Schistosomiasis japonica | Colitis | 0.375 |
| Schistosomiasis japonica | Primary Myelofibrosis | 0.5 |
| Seminoma | Cerebellar Neoplasms | 0.5 |
| Short Bowel Syndrome | Carcinoma, Transitional Cell | 0.5 |
| Short Bowel Syndrome | Malaria, Vivax | 0.5 |
| Skin Diseases | Sepsis | 0.5 |
| Sleep Apnea, Obstructive | Esophagitis | 0.3333333 |
| Sleep Apnea, Obstructive | Osteogenesis Imperfecta | 0.5 |
| Spondylitis, Ankylosing | Glomerulonephritis, IGA | 0.4 |
| Spondylitis, Ankylosing | Peripheral Nervous System Diseases | 0.375 |
| Stomach Neoplasms | Carcinoma, Renal Cell | 0.3386243 |
| Stomach Neoplasms | Glioma | 0.3513514 |
| Stomach Neoplasms | Lung Neoplasms | 0.3684211 |
| Stress Disorders, Post-Traumatic | Liver Diseases, Alcoholic | 0.3333333 |
| Telangiectasia, Hereditary Hemorrhagic | Esophagitis | 0.3333333 |
| Tetralogy of Fallot | Fatigue Syndrome, Chronic | 1 |
| Tetralogy of Fallot | Hearing Loss | 1 |
| Tetralogy of Fallot | Hyperuricemia | 1 |
| Tetralogy of Fallot | Intracranial Aneurysm | 0.3333333 |
| Tetralogy of Fallot | Leukemia, Megakaryocytic, Acute | 0.5 |
| Tetralogy of Fallot | MELAS Syndrome | 1 |
| Tetralogy of Fallot | Metabolic Diseases | 0.5 |
| Thrombocythemia, Hemorrhagic | Myeloproliferative Disorders | 0.3333333 |
| Thrombocytopenia | Angina, Unstable | 0.3333333 |
| Thrombocytopenia | Colorectal Neoplasms, Hereditary Nonpolyposis | 0.5 |
| Thrombocytopenia | Dermatitis, Allergic Contact | 0.3333333 |
| Thrombocytopenia | Encephalitis | 0.3333333 |
| Thrombocytopenia | Fatigue Syndrome, Chronic | 0.5 |
| Thrombocytopenia | Gastritis, Atrophic | 0.5 |
| Thrombocytopenia | Gaucher Disease | 0.5 |
| Thrombocytopenia | Hearing Loss | 0.5 |
| Thrombocytopenia | Hyperuricemia | 0.5 |

| | | |
|---|---|---|
| Thrombocytopenia | Leukemia, Megakaryocytic, Acute | 0.3333333 |
| Thrombocytopenia | MELAS Syndrome | 0.5 |
| Thrombocytopenia | Metabolic Diseases | 0.3333333 |
| Thrombocytopenia | Nephrolithiasis | 0.5 |
| Thrombocytopenia | Tetralogy of Fallot | 0.5 |
| Thrombocytosis | Alcoholism | 0.5 |
| Thrombocytosis | Arthritis, Juvenile Rheumatoid | 0.3333333 |
| Thrombocytosis | Autoimmune Lymphoproliferative Syndrome | 0.3333333 |
| Thrombocytosis | Chlamydia Infections | 0.5 |
| Thrombocytosis | Coronary Restenosis | 0.5 |
| Thrombocytosis | Creutzfeldt-Jakob Syndrome | 0.5 |
| Thrombocytosis | Leprosy | 0.3333333 |
| Thrombocytosis | Meningitis | 0.5 |
| Thrombocytosis | Mycobacterium avium-intracellulare Infection | 0.5 |
| Thrombocytosis | Osteogenesis Imperfecta | 0.3333333 |
| Thrombocytosis | Pulmonary Heart Disease | 0.5 |
| Thrombocytosis | Sleep Apnea, Obstructive | 0.5 |
| Thrombosis | Hemangiosarcoma | 0.4 |
| Thyroiditis, Autoimmune | Colorectal Neoplasms, Hereditary Nonpolyposis | 0.3333333 |
| Thyroiditis, Autoimmune | Gastritis, Atrophic | 0.3333333 |
| Thyroiditis, Autoimmune | Gaucher Disease | 0.3333333 |
| Thyroiditis, Autoimmune | Nephrolithiasis | 0.3333333 |
| Tourette Syndrome | Osteomyelitis | 0.5 |
| Toxoplasmosis | Lymphoma, Large-Cell, Ki-1 | 0.4666667 |
| Uterine Cervical Neoplasms | Carcinoma, Pancreatic Ductal | 0.3383459 |
| Uterine Cervical Neoplasms | Glioma | 0.354067 |
| Uterine Cervical Neoplasms | Osteosarcoma | 0.3405405 |
| Uterine Cervical Neoplasms | Prostatic Neoplasms | 0.3579545 |
| Uterine Neoplasms | Fatigue Syndrome, Chronic | 1 |
| Uterine Neoplasms | Hearing Loss | 1 |
| Uterine Neoplasms | Hyperuricemia | 1 |
| Uterine Neoplasms | Intracranial Aneurysm | 0.3333333 |
| Uterine Neoplasms | Leukemia, Megakaryocytic, Acute | 0.5 |
| Uterine Neoplasms | MELAS Syndrome | 1 |
| Uterine Neoplasms | Metabolic Diseases | 0.5 |
| Uterine Neoplasms | Tetralogy of Fallot | 1 |
| Uterine Neoplasms | Thrombocytopenia | 0.5 |
| Uveitis | Alcoholism | 1 |
| Uveitis | Arthritis, Juvenile Rheumatoid | 0.5 |
| Uveitis | Arthritis, Psoriatic | 0.3333333 |
| Uveitis | Autoimmune Lymphoproliferative Syndrome | 0.5 |
| Uveitis | Chlamydia Infections | 1 |
| Uveitis | Coronary Restenosis | 1 |
| Uveitis | Creutzfeldt-Jakob Syndrome | 1 |
| Uveitis | Leprosy | 0.5 |
| Uveitis | Meningitis | 1 |
| Uveitis | Mycobacterium avium-intracellulare Infection | 1 |
| Uveitis | Peripheral Nervous System Diseases | 0.3333333 |
| Uveitis | Pneumonia | 0.3333333 |
| Uveitis | Pulmonary Heart Disease | 1 |
| Uveitis | Thrombocytosis | 0.5 |
| Uveomeningoencephalitic Syndrome | Alcoholism | 1 |
| Uveomeningoencephalitic Syndrome | Arthritis, Juvenile Rheumatoid | 0.5 |
| Uveomeningoencephalitic Syndrome | Arthritis, Psoriatic | 0.3333333 |
| Uveomeningoencephalitic Syndrome | Autoimmune Lymphoproliferative Syndrome | 0.5 |
| Uveomeningoencephalitic Syndrome | Chlamydia Infections | 1 |

| | | |
|---|---|---|
| Uveomeningoencephalitic Syndrome | Coronary Restenosis | 1 |
| Uveomeningoencephalitic Syndrome | Creutzfeldt-Jakob Syndrome | 1 |
| Uveomeningoencephalitic Syndrome | Leprosy | 0.5 |
| Uveomeningoencephalitic Syndrome | Meningitis | 1 |
| Uveomeningoencephalitic Syndrome | Mycobacterium avium-intracellulare Infection | 1 |
| Uveomeningoencephalitic Syndrome | Peripheral Nervous System Diseases | 0.3333333 |
| Uveomeningoencephalitic Syndrome | Pneumonia | 0.3333333 |
| Uveomeningoencephalitic Syndrome | Pulmonary Heart Disease | 1 |
| Uveomeningoencephalitic Syndrome | Thrombocytosis | 0.5 |
| Uveomeningoencephalitic Syndrome | Uveitis | 1 |
| Varicocele | Arthritis, Psoriatic | 0.3333333 |
| Varicocele | Heart Valve Diseases | 0.5 |
| Varicocele | Polycystic Kidney Diseases | 0.5 |
| Vitiligo | Colorectal Neoplasms, Hereditary Nonpolyposis | 0.3333333 |
| Vitiligo | Gastritis, Atrophic | 0.3333333 |
| Vitiligo | Gaucher Disease | 0.3333333 |
| Vitiligo | Heart Defects, Congenital | 0.3333333 |
| Vitiligo | Nephrolithiasis | 0.3333333 |
| Vitiligo | Sleep Apnea, Obstructive | 0.3333333 |
| Vulvar Neoplasms | Polycystic Kidney Diseases | 0.5 |
| Vulvar Neoplasms | Severe Acute Respiratory Syndrome | 0.3333333 |

| Source | Target | similarity |
|---|---|---|
| Alzheimer Disease | Atherosclerosis | 0.98562754 |
| Alzheimer Disease | Brain Diseases | 0.98842779 |
| Alzheimer Disease | Breast Neoplasms | 0.985996 |
| Alzheimer Disease | Carcinoma, Hepatocellular | 0.98597672 |
| Alzheimer Disease | Carcinoma, Squamous Cell | 0.98399434 |
| Alzheimer Disease | Coronary artery disease | 0.9839721 |
| Alzheimer Disease | Diabetes Mellitus | 0.98970786 |
| Alzheimer Disease | Diabetes Mellitus, Type 2 | 0.991973 |
| Alzheimer Disease | Esophageal Neoplasms | 0.9838278 |
| Alzheimer Disease | Hepatitis B | 0.98594711 |
| Alzheimer Disease | Hepatitis C | 0.98402599 |
| Alzheimer Disease | Hypertension | 0.9877321 |
| Alzheimer Disease | Immune System Diseases | 0.98363692 |
| Alzheimer Disease | Kidney Failure, Chronic | 0.98449754 |
| Alzheimer Disease | Liver Neoplasms | 0.98443263 |
| Alzheimer Disease | Lung Neoplasms | 0.9859086 |
| Alzheimer Disease | Metabolic Syndrome X | 0.98858764 |
| Alzheimer Disease | Multiple Sclerosis | 0.98456408 |
| Alzheimer Disease | Myocardial Infarction | 0.98601264 |
| Alzheimer Disease | Obesity | 0.98794866 |
| Alzheimer Disease | Osteoporosis | 0.9854498 |
| Alzheimer Disease | Prostatic Neoplasms | 0.9851983 |
| Alzheimer Disease | Schizophrenia | 0.98464465 |
| Alzheimer Disease | Stomach Neoplasms | 0.98481312 |
| Anxiety Disorders | Panic Disorder | 0.98454806 |
| Arthritis, Rheumatoid | Diabetes Mellitus, Type 2 | 0.9843674 |
| Arthritis, Rheumatoid | Hepatitis B | 0.98706643 |
| Arthritis, Rheumatoid | Hepatitis C | 0.98632469 |
| Asthma | Breast Neoplasms | 0.98390865 |
| Asthma | Carcinoma, Hepatocellular | 0.98385279 |
| Asthma | Diabetes Mellitus | 0.98671575 |
| Asthma | Diabetes Mellitus, Type 2 | 0.98718208 |
| Asthma | Hepatitis B | 0.98736443 |
| Asthma | Hepatitis C | 0.98536174 |
| Asthma | Hypersensitivity | 0.98483383 |
| Asthma | Hypertension | 0.98619547 |
| Asthma | Lung Neoplasms | 0.98551194 |
| Asthma | Lupus Erythematosus, Systemic | 0.98473574 |
| Asthma | Lymphoma | 0.98406379 |
| Asthma | Multiple Sclerosis | 0.98543879 |
| Atherosclerosis | Carcinoma, Hepatocellular | 0.98366358 |
| Atherosclerosis | Carcinoma, Squamous Cell | 0.9837216 |
| Atherosclerosis | Coronary artery disease | 0.99185535 |
| Atherosclerosis | Diabetes Mellitus | 0.98753417 |
| Atherosclerosis | Diabetes Mellitus, Type 2 | 0.988395 |
| Atherosclerosis | Heart Diseases | 0.98527068 |
| Atherosclerosis | Hepatitis B | 0.98468887 |
| Atherosclerosis | Hepatitis C | 0.98424177 |
| Atherosclerosis | Hypertension | 0.98652581 |
| Atherosclerosis | Ischemia | 0.98578906 |
| Atherosclerosis | Kidney Failure, Chronic | 0.98496804 |
| Atherosclerosis | Liver Cirrhosis | 0.98507848 |
| Atherosclerosis | Metabolic Syndrome X | 0.98661013 |
| Atherosclerosis | Myocardial Infarction | 0.98890541 |
| Atherosclerosis | Obesity | 0.98590874 |

| | | |
|---|---|---|
| Atherosclerosis | Pre-Eclampsia | 0.98385213 |
| Bipolar Disorder | Schizophrenia | 0.98375155 |
| Brain Diseases | Diabetes Mellitus | 0.98627754 |
| Brain Diseases | Diabetes Mellitus, Type 2 | 0.98682982 |
| Brain Diseases | Hypertension | 0.98493099 |
| Brain Diseases | Obesity | 0.98452815 |
| Breast Neoplasms | Carcinoma, Hepatocellular | 0.98928784 |
| Breast Neoplasms | Carcinoma, Squamous Cell | 0.98742686 |
| Breast Neoplasms | Colorectal Neoplasms | 0.98623983 |
| Breast Neoplasms | Diabetes Mellitus | 0.98813999 |
| Breast Neoplasms | Diabetes Mellitus, Type 2 | 0.98822106 |
| Breast Neoplasms | Esophageal Neoplasms | 0.98775371 |
| Breast Neoplasms | Glioma | 0.9838862 |
| Breast Neoplasms | Hepatitis B | 0.98741511 |
| Breast Neoplasms | Hepatitis C | 0.98637204 |
| Breast Neoplasms | Kidney Failure, Chronic | 0.98746513 |
| Breast Neoplasms | Liver Neoplasms | 0.98614945 |
| Breast Neoplasms | Lung Neoplasms | 0.99041989 |
| Breast Neoplasms | Lymphoma | 0.98595912 |
| Breast Neoplasms | Multiple Sclerosis | 0.98443869 |
| Breast Neoplasms | Obesity | 0.98548617 |
| Breast Neoplasms | Ovarian Neoplasms | 0.99006673 |
| Breast Neoplasms | Pancreatic Neoplasms | 0.98627686 |
| Breast Neoplasms | Prostatic Neoplasms | 0.98675422 |
| Breast Neoplasms | Pulmonary Disease, Chronic Obstructive | 0.98594764 |
| Breast Neoplasms | Stomach Neoplasms | 0.98941171 |
| Breast Neoplasms | Urinary Bladder Neoplasms | 0.98818813 |
| Carcinoma, Hepatocellular | Carcinoma, Squamous Cell | 0.9859452 |
| Carcinoma, Hepatocellular | Colorectal Neoplasms | 0.98403647 |
| Carcinoma, Hepatocellular | Diabetes Mellitus | 0.98731845 |
| Carcinoma, Hepatocellular | Diabetes Mellitus, Type 2 | 0.98772025 |
| Carcinoma, Hepatocellular | Esophageal Neoplasms | 0.98582449 |
| Carcinoma, Hepatocellular | Hepatitis B | 0.98546156 |
| Carcinoma, Hepatocellular | Hepatitis C | 0.9842457 |
| Carcinoma, Hepatocellular | Hypertension | 0.98476043 |
| Carcinoma, Hepatocellular | Kidney Failure, Chronic | 0.98585294 |
| Carcinoma, Hepatocellular | Liver Neoplasms | 0.98566199 |
| Carcinoma, Hepatocellular | Lung Neoplasms | 0.98853629 |
| Carcinoma, Hepatocellular | Lymphoma | 0.98459665 |
| Carcinoma, Hepatocellular | Metabolic Syndrome X | 0.9837493 |
| Carcinoma, Hepatocellular | Multiple Sclerosis | 0.9836219 |
| Carcinoma, Hepatocellular | Obesity | 0.98541108 |
| Carcinoma, Hepatocellular | Pulmonary Disease, Chronic Obstructive | 0.98431585 |
| Carcinoma, Hepatocellular | Stomach Neoplasms | 0.98682325 |
| Carcinoma, Non-Small-Cell Lung | Lung Neoplasms | 0.98729559 |
| Carcinoma, Non-Small-Cell Lung | Pulmonary Disease, Chronic Obstructive | 0.98396047 |
| Carcinoma, Squamous Cell | Colorectal Neoplasms | 0.98447538 |
| Carcinoma, Squamous Cell | Diabetes Mellitus | 0.98770947 |
| Carcinoma, Squamous Cell | Diabetes Mellitus, Type 2 | 0.98702263 |
| Carcinoma, Squamous Cell | Esophageal Neoplasms | 0.98855653 |
| Carcinoma, Squamous Cell | Kidney Failure, Chronic | 0.98493859 |
| Carcinoma, Squamous Cell | Liver Neoplasms | 0.98453358 |
| Carcinoma, Squamous Cell | Lung Neoplasms | 0.98904803 |
| Carcinoma, Squamous Cell | Lymphoma | 0.98375845 |
| Carcinoma, Squamous Cell | Metabolic Syndrome X | 0.98366132 |
| Carcinoma, Squamous Cell | Obesity | 0.98417183 |

| | | |
|---|---|---|
| Carcinoma, Squamous Cell | Ovarian Neoplasms | 0.98795 |
| Carcinoma, Squamous Cell | Pulmonary Disease, Chronic Obstructive | 0.98408943 |
| Carcinoma, Squamous Cell | Stomach Neoplasms | 0.98663767 |
| Colitis, Ulcerative | Inflammatory Bowel Diseases | 0.98464622 |
| Colorectal Neoplasms | Diabetes Mellitus, Type 2 | 0.98471294 |
| Colorectal Neoplasms | Esophageal Neoplasms | 0.98372269 |
| Colorectal Neoplasms | Lung Neoplasms | 0.98710033 |
| Colorectal Neoplasms | Stomach Neoplasms | 0.98584402 |
| Coronary artery disease | Diabetes Mellitus | 0.98774788 |
| Coronary artery disease | Diabetes Mellitus, Type 2 | 0.98830804 |
| Coronary artery disease | Heart Diseases | 0.98683927 |
| Coronary artery disease | Hypertension | 0.98668095 |
| Coronary artery disease | Ischemia | 0.98718149 |
| Coronary artery disease | Metabolic Syndrome X | 0.98594584 |
| Coronary artery disease | Myocardial Infarction | 0.98939826 |
| Coronary artery disease | Obesity | 0.98435113 |
| Cystic Fibrosis | Diabetes Mellitus | 0.98374554 |
| Cystic Fibrosis | Hepatitis B | 0.98388221 |
| Diabetes Mellitus | Diabetes Mellitus, Type 2 | 0.99469607 |
| Diabetes Mellitus | Esophageal Neoplasms | 0.98606355 |
| Diabetes Mellitus | Glioma | 0.98442763 |
| Diabetes Mellitus | Heart Diseases | 0.98443804 |
| Diabetes Mellitus | Hepatitis B | 0.98780111 |
| Diabetes Mellitus | Hepatitis C | 0.98663583 |
| Diabetes Mellitus | Hypertension | 0.99031488 |
| Diabetes Mellitus | Kidney Failure, Chronic | 0.98926471 |
| Diabetes Mellitus | Liver Cirrhosis | 0.98531768 |
| Diabetes Mellitus | Liver Neoplasms | 0.98617211 |
| Diabetes Mellitus | Lung Neoplasms | 0.98845898 |
| Diabetes Mellitus | Lymphoma | 0.98658381 |
| Diabetes Mellitus | Lymphoma, Non-Hodgkin | 0.98379638 |
| Diabetes Mellitus | Melanoma | 0.98425359 |
| Diabetes Mellitus | Metabolic Syndrome X | 0.98983602 |
| Diabetes Mellitus | Multiple Sclerosis | 0.98596851 |
| Diabetes Mellitus | Myocardial Infarction | 0.98899695 |
| Diabetes Mellitus | Obesity | 0.99123846 |
| Diabetes Mellitus | Osteoporosis | 0.98514184 |
| Diabetes Mellitus | Ovarian Neoplasms | 0.98369593 |
| Diabetes Mellitus | Prostatic Neoplasms | 0.98447682 |
| Diabetes Mellitus | Pulmonary Disease, Chronic Obstructive | 0.98422339 |
| Diabetes Mellitus | Schizophrenia | 0.98516946 |
| Diabetes Mellitus | Stomach Neoplasms | 0.98502862 |
| Diabetes Mellitus | Urinary Bladder Neoplasms | 0.9841696 |
| Diabetes Mellitus, Type 2 | Esophageal Neoplasms | 0.98681709 |
| Diabetes Mellitus, Type 2 | Glioma | 0.98596246 |
| Diabetes Mellitus, Type 2 | Heart Diseases | 0.985219 |
| Diabetes Mellitus, Type 2 | Hepatitis B | 0.98832558 |
| Diabetes Mellitus, Type 2 | Hepatitis C | 0.98778228 |
| Diabetes Mellitus, Type 2 | Hypertension | 0.99235249 |
| Diabetes Mellitus, Type 2 | Immune System Diseases | 0.9855628 |
| Diabetes Mellitus, Type 2 | Ischemia | 0.98519213 |
| Diabetes Mellitus, Type 2 | Kidney Failure, Chronic | 0.98889426 |
| Diabetes Mellitus, Type 2 | Liver Cirrhosis | 0.98589895 |
| Diabetes Mellitus, Type 2 | Liver Neoplasms | 0.98575674 |
| Diabetes Mellitus, Type 2 | Lung Neoplasms | 0.98941364 |
| Diabetes Mellitus, Type 2 | Lymphoma | 0.98675873 |

| | | |
|---|---|---|
| Diabetes Mellitus, Type 2 | Lymphoma, Non-Hodgkin | 0.98392486 |
| Diabetes Mellitus, Type 2 | Melanoma | 0.98551011 |
| Diabetes Mellitus, Type 2 | Metabolic Syndrome X | 0.99191631 |
| Diabetes Mellitus, Type 2 | Multiple Sclerosis | 0.98787046 |
| Diabetes Mellitus, Type 2 | Myocardial Infarction | 0.98820351 |
| Diabetes Mellitus, Type 2 | Obesity | 0.99167324 |
| Diabetes Mellitus, Type 2 | Osteoporosis | 0.98552305 |
| Diabetes Mellitus, Type 2 | Ovarian Neoplasms | 0.98438309 |
| Diabetes Mellitus, Type 2 | Prostatic Neoplasms | 0.98644046 |
| Diabetes Mellitus, Type 2 | Pulmonary Disease, Chronic Obstructive | 0.98398821 |
| Diabetes Mellitus, Type 2 | Schizophrenia | 0.98580679 |
| Diabetes Mellitus, Type 2 | Stomach Neoplasms | 0.98641347 |
| Diabetes Mellitus, Type 2 | Urinary Bladder Neoplasms | 0.98382826 |
| Endometrial Neoplasms | Esophageal Neoplasms | 0.98379492 |
| Endometrial Neoplasms | Ovarian Neoplasms | 0.98598974 |
| Endometrial Neoplasms | Stomach Neoplasms | 0.98381219 |
| Esophageal Neoplasms | Glioma | 0.98417034 |
| Esophageal Neoplasms | Kidney Failure, Chronic | 0.98488328 |
| Esophageal Neoplasms | Leukemia, B-Cell, Chronic | 0.983749 |
| Esophageal Neoplasms | Liver Neoplasms | 0.98465156 |
| Esophageal Neoplasms | Lung Neoplasms | 0.98858184 |
| Esophageal Neoplasms | Lymphoma | 0.98550773 |
| Esophageal Neoplasms | Multiple Sclerosis | 0.98389392 |
| Esophageal Neoplasms | Ovarian Neoplasms | 0.98644411 |
| Esophageal Neoplasms | Prostatic Neoplasms | 0.98379283 |
| Esophageal Neoplasms | Pulmonary Disease, Chronic Obstructive | 0.98521387 |
| Esophageal Neoplasms | Stomach Neoplasms | 0.98616473 |
| Esophageal Neoplasms | Urinary Bladder Neoplasms | 0.98596149 |
| Glioma | Hepatitis B | 0.98432304 |
| Glioma | Hepatitis C | 0.98375806 |
| Glioma | Leukemia, Lymphocytic | 0.98635044 |
| Glioma | Leukemia, Lymphocytic, Acute | 0.98622156 |
| Glioma | Leukemia, Myelocytic, Acute | 0.98735386 |
| Glioma | Leukemia, Myeloid | 0.98444253 |
| Glioma | Lung Neoplasms | 0.98628562 |
| Glioma | Lymphoma | 0.98744839 |
| Glioma | Meningioma | 0.98569326 |
| Glioma | Stomach Neoplasms | 0.98393107 |
| Heart Diseases | Hypertension | 0.98514483 |
| Heart Diseases | Myocardial Infarction | 0.98418119 |
| Hepatitis B | Hepatitis C | 0.99424676 |
| Hepatitis B | Hypertension | 0.98474742 |
| Hepatitis B | Kidney Failure, Chronic | 0.98502951 |
| Hepatitis B | Liver Cirrhosis | 0.98438928 |
| Hepatitis B | Liver Neoplasms | 0.98562112 |
| Hepatitis B | Lung Neoplasms | 0.98634105 |
| Hepatitis B | Lupus Erythematosus, Systemic | 0.98661302 |
| Hepatitis B | Lymphoma | 0.98743847 |
| Hepatitis B | Multiple Sclerosis | 0.98498974 |
| Hepatitis B | Myocardial Infarction | 0.98421756 |
| Hepatitis B | Obesity | 0.9842841 |
| Hepatitis C | Hypertension | 0.98376861 |
| Hepatitis C | Kidney Failure, Chronic | 0.98396104 |
| Hepatitis C | Leukemia, Lymphocytic, Acute | 0.98385556 |
| Hepatitis C | Liver Neoplasms | 0.98543883 |
| Hepatitis C | Lung Neoplasms | 0.98509351 |

| | | |
|---|---|---|
| Hepatitis C | Lupus Erythematosus, Systemic | 0.98569311 |
| Hepatitis C | Lymphoma | 0.98575289 |
| Hepatitis C | Multiple Sclerosis | 0.9851868 |
| Hepatitis C | Myocardial Infarction | 0.98385469 |
| Hodgkin Disease | Lymphoma | 0.98369552 |
| Hypertension | Ischemia | 0.98395914 |
| Hypertension | Kidney Failure, Chronic | 0.98486644 |
| Hypertension | Liver Cirrhosis | 0.9837056 |
| Hypertension | Lung Neoplasms | 0.98580912 |
| Hypertension | Metabolic Syndrome X | 0.9877571 |
| Hypertension | Multiple Sclerosis | 0.98469119 |
| Hypertension | Myocardial Infarction | 0.98849478 |
| Hypertension | Obesity | 0.98972862 |
| Hypertension | Schizophrenia | 0.98426184 |
| Ischemia | Myocardial Infarction | 0.98365601 |
| Ischemia | Obesity | 0.98361726 |
| Kidney Failure, Chronic | Liver Neoplasms | 0.98538638 |
| Kidney Failure, Chronic | Lung Neoplasms | 0.9882643 |
| Kidney Failure, Chronic | Lymphoma | 0.9853608 |
| Kidney Failure, Chronic | Metabolic Syndrome X | 0.9856025 |
| Kidney Failure, Chronic | Obesity | 0.98618486 |
| Kidney Failure, Chronic | Ovarian Neoplasms | 0.98383828 |
| Kidney Failure, Chronic | Pulmonary Disease, Chronic Obstructive | 0.98381058 |
| Kidney Failure, Chronic | Stomach Neoplasms | 0.98610956 |
| Kidney Failure, Chronic | Urinary Bladder Neoplasms | 0.98512736 |
| Leukemia, B-Cell, Chronic | Lung Neoplasms | 0.98906243 |
| Leukemia, B-Cell, Chronic | Lymphoma | 0.98540092 |
| Leukemia, B-Cell, Chronic | Stomach Neoplasms | 0.9844053 |
| Leukemia, Lymphocytic | Leukemia, Lymphocytic, Acute | 0.99105228 |
| Leukemia, Lymphocytic | Leukemia, Myelocytic, Acute | 0.98779971 |
| Leukemia, Lymphocytic | Leukemia, Myeloid | 0.98498096 |
| Leukemia, Lymphocytic | Lymphoma | 0.98568274 |
| Leukemia, Lymphocytic, Acute | Leukemia, Myelocytic, Acute | 0.98794955 |
| Leukemia, Lymphocytic, Acute | Leukemia, Myeloid | 0.98416459 |
| Leukemia, Lymphocytic, Acute | Lymphoma | 0.98501226 |
| Leukemia, Myelocytic, Acute | Leukemia, Myeloid | 0.98813992 |
| Leukemia, Myelocytic, Acute | Lymphoma | 0.98581247 |
| Leukemia, Myelocytic, Acute | Meningioma | 0.98467326 |
| Leukemia, Myeloid | Lymphoma | 0.98444336 |
| Liver Cirrhosis | Lung Neoplasms | 0.98360323 |
| Liver Cirrhosis | Myocardial Infarction | 0.98381838 |
| Liver Neoplasms | Lung Neoplasms | 0.98716106 |
| Liver Neoplasms | Obesity | 0.98482561 |
| Liver Neoplasms | Pulmonary Disease, Chronic Obstructive | 0.9854322 |
| Liver Neoplasms | Stomach Neoplasms | 0.98517454 |
| Liver Neoplasms | Urinary Bladder Neoplasms | 0.98390591 |
| Lung Neoplasms | Lymphoma | 0.98720749 |
| Lung Neoplasms | Lymphoma, Non-Hodgkin | 0.98441362 |
| Lung Neoplasms | Metabolic Syndrome X | 0.98432669 |
| Lung Neoplasms | Multiple Sclerosis | 0.98488523 |
| Lung Neoplasms | Obesity | 0.98639819 |
| Lung Neoplasms | Ovarian Neoplasms | 0.9873794 |
| Lung Neoplasms | Pancreatic Neoplasms | 0.98442212 |
| Lung Neoplasms | Prostatic Neoplasms | 0.98530727 |
| Lung Neoplasms | Pulmonary Disease, Chronic Obstructive | 0.98953975 |
| Lung Neoplasms | Stomach Neoplasms | 0.98849811 |

| | | |
|---|---|---|
| Lung Neoplasms | Urinary Bladder Neoplasms | 0.98865363 |
| Lupus Erythematosus, Systemic | Lymphoma | 0.98420509 |
| Lymphoma | Lymphoma, Non-Hodgkin | 0.99013943 |
| Lymphoma | Multiple Myeloma | 0.98490546 |
| Lymphoma | Stomach Neoplasms | 0.98505788 |
| Lymphoma, B-Cell | Lymphoma, Large-Cell, Diffuse | 0.98633325 |
| Lymphoma, Non-Hodgkin | Multiple Myeloma | 0.98473238 |
| Metabolic Syndrome X | Myocardial Infarction | 0.9845326 |
| Metabolic Syndrome X | Obesity | 0.98841429 |
| Metabolic Syndrome X | Osteoporosis | 0.98586218 |
| Multiple Sclerosis | Obesity | 0.98450463 |
| Obesity | Osteoporosis | 0.98370033 |
| Obesity | Polycystic Ovary Syndrome | 0.98482318 |
| Obesity | Prostatic Neoplasms | 0.98429573 |
| Obesity | Schizophrenia | 0.98484432 |
| Ovarian Neoplasms | Pulmonary Disease, Chronic Obstructive | 0.98383284 |
| Ovarian Neoplasms | Stomach Neoplasms | 0.98742354 |
| Ovarian Neoplasms | Urinary Bladder Neoplasms | 0.98464459 |
| Pulmonary Disease, Chronic Obstructive | Stomach Neoplasms | 0.98466255 |
| Pulmonary Disease, Chronic Obstructive | Urinary Bladder Neoplasms | 0.98766118 |
| Stomach Neoplasms | Urinary Bladder Neoplasms | 0.98465646 |

**Supplementary Table 2:** The mpDisNet-predicted 100 comorbid disease pairs.

| Source | Target | similarity |
|---|---|---|
| Alzheimer Disease | Long QT Syndrome | 0.88377895 |
| Anemia, Sickle Cell | Cerebral Infarction | 0.959475536 |
| Anemia, Sickle Cell | Mental Retardation | 0.92063305 |
| Anemia, Sickle Cell | Mucocutaneous Lymph Node Syndrome | 0.937345709 |
| Arthritis, Rheumatoid | Friedreich Ataxia | 0.883647145 |
| Arthritis, Rheumatoid | Long QT Syndrome | 0.871775004 |
| Arthritis, Rheumatoid | Multiple Endocrine Neoplasia Type 1 | 0.908967166 |
| Arthritis, Rheumatoid | Osteogenesis Imperfecta | 0.898680006 |
| Arthritis, Rheumatoid | Telangiectasia, Hereditary Hemorrhagic | 0.865650556 |
| Arthritis, Rheumatoid | Thalassemia | 0.916529494 |
| Asthma | Multiple Endocrine Neoplasia Type 1 | 0.910167409 |
| Asthma | Telangiectasia, Hereditary Hemorrhagic | 0.867767363 |
| Asthma | Thalassemia | 0.921268812 |
| Autistic Disorder | DiGeorge Syndrome | 0.876359596 |
| Autistic Disorder | Long QT Syndrome | 0.884154918 |
| Autoimmune Lymphoproliferative Syndrome | Bipolar Disorder | 0.862429743 |
| Autoimmune Lymphoproliferative Syndrome | Cataract | 0.846010311 |
| Autoimmune Lymphoproliferative Syndrome | Celiac Disease | 0.869614916 |
| Autoimmune Lymphoproliferative Syndrome | Crohn Disease | 0.877102919 |
| Bipolar Disorder | Friedreich Ataxia | 0.895441539 |
| Bipolar Disorder | Telangiectasia, Hereditary Hemorrhagic | 0.844632261 |
| Bipolar Disorder | Thalassemia | 0.908394697 |
| Brain Neoplasms | Long QT Syndrome | 0.877148389 |
| Brain Neoplasms | Multiple Endocrine Neoplasia Type 1 | 0.912947692 |
| Brain Neoplasms | Osteogenesis Imperfecta | 0.906857593 |
| Brain Neoplasms | Telangiectasia, Hereditary Hemorrhagic | 0.86282811 |
| Brain Neoplasms | Thalassemia | 0.913234913 |
| Breast Neoplasms | Long QT Syndrome | 0.878023109 |
| Breast Neoplasms | Thalassemia | 0.919227692 |
| Burkitt Lymphoma | Long QT Syndrome | 0.795018148 |
| Cataract | Friedreich Ataxia | 0.862387952 |
| Cataract | Long QT Syndrome | 0.846656727 |
| Cataract | Multiple Endocrine Neoplasia Type 1 | 0.891000081 |
| Cataract | Osteogenesis Imperfecta | 0.885094657 |
| Cataract | Retinitis Pigmentosa | 0.88522725 |
| Cataract | Telangiectasia, Hereditary Hemorrhagic | 0.83093475 |
| Cataract | Thalassemia | 0.881922676 |
| Celiac Disease | Down Syndrome | 0.950323165 |
| Celiac Disease | Long QT Syndrome | 0.867790085 |
| Celiac Disease | Osteogenesis Imperfecta | 0.895050164 |
| Celiac Disease | Retinitis Pigmentosa | 0.883439596 |
| Celiac Disease | Telangiectasia, Hereditary Hemorrhagic | 0.863223629 |
| Celiac Disease | Thalassemia | 0.908074923 |
| Cerebral Infarction | Friedreich Ataxia | 0.893480417 |
| Cerebral Infarction | Long QT Syndrome | 0.868903821 |
| Cerebral Infarction | Multiple Endocrine Neoplasia Type 1 | 0.897789769 |
| Cerebral Infarction | Osteogenesis Imperfecta | 0.901453881 |

| | | |
|---|---|---|
| Cerebral Infarction | Retinitis Pigmentosa | 0.884800732 |
| Cerebral Infarction | Telangiectasia, Hereditary Hemorrhagic | 0.859235693 |
| Cerebral Infarction | Thalassemia | 0.931756901 |
| Colorectal Neoplasms | Telangiectasia, Hereditary Hemorrhagic | 0.871068863 |
| Colorectal Neoplasms | Thalassemia | 0.913359088 |
| Crohn Disease | Cystic Fibrosis | 0.948487942 |
| Crohn Disease | Familial Mediterranean Fever | 0.903763634 |
| Crohn Disease | Long QT Syndrome | 0.877117859 |
| Crohn Disease | Retinitis Pigmentosa | 0.858360243 |
| Crohn Disease | Telangiectasia, Hereditary Hemorrhagic | 0.855701262 |
| Crohn Disease | Thalassemia | 0.914728173 |
| Cystic Fibrosis | Mental Retardation | 0.946905933 |
| DiGeorge Syndrome | Mental Retardation | 0.880473087 |
| DiGeorge Syndrome | Osteoarthritis | 0.877733149 |
| DiGeorge Syndrome | Schizophrenia | 0.884501597 |
| Down Syndrome | Mental Retardation | 0.946847286 |
| Epilepsy | Long QT Syndrome | 0.896304609 |
| Epilepsy | Osteogenesis Imperfecta | 0.881573118 |
| Epilepsy | Telangiectasia, Hereditary Hemorrhagic | 0.847149935 |
| Epilepsy | Thalassemia | 0.900623545 |
| Friedreich Ataxia | Glaucoma | 0.886288654 |
| Friedreich Ataxia | Mental Retardation | 0.871810639 |
| Glaucoma | Long QT Syndrome | 0.875035008 |
| Glaucoma | Multiple Endocrine Neoplasia Type 1 | 0.894753529 |
| Gout | Long QT Syndrome | 0.861469602 |
| Gout | Retinitis Pigmentosa | 0.846508532 |
| Gout | Telangiectasia, Hereditary Hemorrhagic | 0.846711676 |
| Gout | Thalassemia | 0.871380227 |
| Kidney Neoplasms | Long QT Syndrome | 0.852654094 |
| Kidney Neoplasms | Telangiectasia, Hereditary Hemorrhagic | 0.848121554 |
| Kidney Neoplasms | Thalassemia | 0.888938627 |
| Long QT Syndrome | Lung Neoplasms | 0.879435196 |
| Long QT Syndrome | Mental Retardation | 0.900263417 |
| Long QT Syndrome | Mucocutaneous Lymph Node Syndrome | 0.869763547 |
| Long QT Syndrome | Osteoarthritis | 0.868596049 |
| Long QT Syndrome | Prostatic Neoplasms | 0.873711733 |
| Long QT Syndrome | Psoriasis | 0.876188572 |
| Long QT Syndrome | Schizophrenia | 0.889944888 |
| Long QT Syndrome | Uterine Neoplasms | 0.894333596 |
| Lung Neoplasms | Thalassemia | 0.920317475 |
| Mental Retardation | Osteogenesis Imperfecta | 0.872733159 |
| Mental Retardation | Retinitis Pigmentosa | 0.864296155 |
| Mental Retardation | Thalassemia | 0.91636092 |
| Mucocutaneous Lymph Node Syndrome | Thalassemia | 0.909956341 |
| Multiple Endocrine Neoplasia Type 1 | Stomach Neoplasms | 0.918279365 |
| Osteoarthritis | Telangiectasia, Hereditary Hemorrhagic | 0.857912694 |
| Osteoarthritis | Thalassemia | 0.904729914 |
| Prostatic Neoplasms | Thalassemia | 0.917720917 |

| | | |
|---|---|---|
| Psoriasis | Telangiectasia, Hereditary Hemorrhagic | 0.861097381 |
| Psoriasis | Thalassemia | 0.90872626 |
| Telangiectasia, Hereditary Hemorrhagic | Uterine Neoplasms | 0.868010995 |
| Bipolar Disorder | Long QT Syndrome | 0.884813424 |
| Bipolar Disorder | Osteogenesis Imperfecta | 0.885962878 |

**Supplementary Table 3:** The 66 clinically reported disease-disease pairs.

| Disease1 | mesh.id1 | Disease2 | mesh.id2 |
|---|---|---|---|
| Alzheimer Disease | D000544 | Schizophrenia | D012559 |
| Alzheimer Disease | D000544 | Mental Retardation | D008607 |
| Alzheimer Disease | D000544 | Brain Diseases | D001927 |
| Alzheimer Disease | D000544 | Bipolar Disorder | D001714 |
| Alzheimer Disease | D000544 | Diabetic Nephropathies | D003928 |
| Alzheimer Disease | D000544 | Epilepsy | D004827 |
| Alzheimer Disease | D000544 | Diabetes Mellitus | D003920 |
| Anemia | D000740 | Endometriosis | D004715 |
| Asthma | D001249 | Arthritis, Rheumatoid | D001172 |
| Asthma | D001249 | Diabetes Mellitus | D003920 |
| Asthma | D001249 | Obesity | D009765 |
| Asthma | D001249 | Pulmonary Disease, Chronic Obstructive | D029424 |
| Bipolar Disorder | D001714 | Schizophrenia | D012559 |
| Brain Diseases | D001927 | Lipid Metabolism, Inborn Errors | D008052 |
| Brain Diseases | D001927 | Diabetes Mellitus | D003920 |
| Cardiomyopathy, Dilated | D002311 | Hyperlipoproteinemia Type III | D006952 |
| Cardiomyopathy, Dilated | D002311 | Diabetic Nephropathies | D003928 |
| Cardiomyopathy, Dilated | D002311 | Myocardial Infarction | D009203 |
| Cardiomyopathy, Dilated | D002311 | Diabetes Mellitus | D003920 |
| Cardiomyopathy, Dilated | D002311 | Lipid Metabolism, Inborn Errors | D008052 |
| Cardiomyopathy, Dilated | D002311 | Obesity | D009765 |
| Pulmonary Disease, Chronic Obstructive | D029424 | Arthritis, Rheumatoid | D001172 |
| Pulmonary Disease, Chronic Obstructive | D029424 | Myocardial Infarction | D009203 |
| Diabetes Mellitus | D003920 | Obesity | D009765 |
| Diabetes Mellitus | D003920 | Lipid Metabolism, Inborn Errors | D008052 |
| Diabetic Nephropathies | D003928 | Myocardial Infarction | D009203 |
| Diabetic Nephropathies | D003928 | Diabetes Mellitus | D003920 |
| Diabetic Nephropathies | D003928 | Lipid Metabolism, Inborn Errors | D008052 |
| Diabetic Nephropathies | D003928 | Obesity | D009765 |
| Endometriosis | D004715 | Arthritis, Rheumatoid | D001172 |
| Epilepsy | D004827 | Multiple Sclerosis | D009103 |
| Epilepsy | D004827 | Brain Diseases | D001927 |
| Epilepsy | D004827 | Schizophrenia | D012559 |
| Huntington Disease | D006816 | Mental Retardation | D008607 |
| Huntington Disease | D006816 | Epilepsy | D004827 |
| Huntington Disease | D006816 | Diabetic Nephropathies | D003928 |
| Huntington Disease | D006816 | Bipolar Disorder | D001714 |

| | | | |
|---|---|---|---|
| Huntington Disease | D006816 | Schizophrenia | D012559 |
| Hyperlipoproteinemia Type III | D006952 | Diabetes Mellitus | D003920 |
| Hyperlipoproteinemia Type III | D006952 | Lipid Metabolism, Inborn Errors | D008052 |
| Hyperlipoproteinemia Type III | D006952 | Myocardial Infarction | D009203 |
| Hyperlipoproteinemia Type III | D006952 | Obesity | D009765 |
| Hyperlipoproteinemia Type III | D006952 | Diabetic Nephropathies | D003928 |
| Hyperlipoproteinemia Type III | D006952 | Brain Diseases | D001927 |
| Infertility, Male | D007248 | Obesity | D009765 |
| Infertility, Male | D007248 | Hyperlipoproteinemia Type III | D006952 |
| Infertility, Male | D007248 | Cystic Fibrosis | D003550 |
| Infertility, Male | D007248 | Diabetic Nephropathies | D003928 |
| Lipid Metabolism, Inborn Errors | D008052 | Obesity | D009765 |
| Malaria, Cerebral | D016779 | Epilepsy | D004827 |
| Malaria, Cerebral | D016779 | Anemia | D000740 |
| Mental Retardation | D008607 | Bipolar Disorder | D001714 |
| Mental Retardation | D008607 | Schizophrenia | D012559 |
| Multiple Sclerosis | D009103 | Diabetes Mellitus | D003920 |
| Myocardial Infarction | D009203 | Diabetes Mellitus | D003920 |
| Myocardial Infarction | D009203 | Obesity | D009765 |
| Myocardial Infarction | D009203 | Lipid Metabolism, Inborn Errors | D008052 |
| Polycystic Kidney Diseases | D007690 | Diabetes Mellitus | D003920 |
| Polycystic Ovary Syndrome | D011085 | Obesity | D009765 |
| Polycystic Ovary Syndrome | D011085 | Diabetes Mellitus | D003920 |
| Polycystic Ovary Syndrome | D011085 | Diabetic Nephropathies | D003928 |
| Polycystic Ovary Syndrome | D011085 | Myocardial Infarction | D009203 |
| Polycystic Ovary Syndrome | D011085 | Lipid Metabolism, Inborn Errors | D008052 |
| Arthritis, Rheumatoid | D001172 | Diabetes Mellitus | D003920 |
| Sarcoidosis, Pulmonary | D017565 | Cardiomyopathy, Dilated | D002311 |
| Sarcoidosis, Pulmonary | D017565 | Pulmonary Disease, Chronic Obstructive | D029424 |

**Supplementary Table 4:** The 52 new clinically reported disease-disease pairs.

| disease1 | mesh.id1 | disease2 | mesh.id2 |
|---|---|---|---|
| Muscular Atrophy, Spinal | D009134 | Adenoma | D000236 |
| Adrenal Cortex Neoplasms | D000306 | Arthritis | D001168 |
| Asthma | D001249 | Arthritis, Rheumatoid | D001172 |
| Endometriosis | D004715 | Arthritis, Rheumatoid | D001172 |
| Pulmonary Disease, Chronic Obstructive | D029424 | Arthritis, Rheumatoid | D001172 |
| Alzheimer Disease | D000544 | Bipolar Disorder | D001714 |
| Huntington Disease | D006816 | Bipolar Disorder | D001714 |
| Lymphoma, Non-Hodgkin | D008228 | Carcinoma, Renal Cell | D002292 |
| Osteoporosis | D010024 | Cardiomyopathies | D009202 |
| Ovarian Diseases | D010049 | Cardiomyopathy, Hypertrophic | D002312 |
| Infertility, Male | D007248 | Cystic Fibrosis | D003550 |
| Alzheimer Disease | D000544 | Diabetes Mellitus | D003920 |
| Arthritis, Rheumatoid | D001172 | Diabetes Mellitus | D003920 |
| Asthma | D001249 | Diabetes Mellitus | D003920 |
| Cardiomyopathy, Dilated | D002311 | Diabetes Mellitus | D003920 |
| Multiple Sclerosis | D009103 | Diabetes Mellitus | D003920 |
| Myocardial Infarction | D009203 | Diabetes Mellitus | D003920 |
| Polycystic Kidney Diseases | D007690 | Diabetes Mellitus | D003920 |
| Polycystic Ovary Syndrome | D011085 | Diabetes Mellitus | D003920 |
| Anemia | D000740 | Endometriosis | D004715 |
| Sjogren's Syndrome | D012859 | Glioblastoma | D005909 |
| Dengue Hemorrhagic Fever | D019595 | Hepatitis E | D016751 |
| Atherosclerosis | D050197 | Influenza, Human | D007251 |
| Atherosclerosis | D050197 | Ischemia | D007511 |
| Adenoma | D000236 | Lymphoma, Non-Hodgkin | D008228 |
| Thyroid Neoplasms | D013964 | Mastocytosis, Systemic | D034721 |
| Carcinoma, Renal Cell | D002292 | Meningioma | D008579 |
| Long QT Syndrome | D008133 | Meningioma | D008579 |
| Encephalitis | D004660 | Meningitis | D008581 |
| Pneumonia | D011014 | Meningitis | D008581 |
| Cardiomyopathy, Dilated | D002311 | Myocardial Infarction | D009203 |
| Polycystic Ovary Syndrome | D011085 | Myocardial Infarction | D009203 |
| Pulmonary Disease, Chronic Obstructive | D029424 | Myocardial Infarction | D009203 |
| Peritonitis | D010538 | Narcolepsy | D009290 |
| Asthma | D001249 | Obesity | D009765 |
| Cardiomyopathy, Dilated | D002311 | Obesity | D009765 |
| Diabetes Mellitus | D003920 | Obesity | D009765 |
| Infertility, Male | D007248 | Obesity | D009765 |
| Myocardial Infarction | D009203 | Obesity | D009765 |
| Polycystic Ovary Syndrome | D011085 | Obesity | D009765 |
| Thalassemia | D013789 | Ovarian Neoplasms | D010051 |
| Nephrolithiasis | D053040 | Pemphigus | D010392 |
| Autistic Disorder | D001321 | Peripheral Nervous System Dise | D010523 |
| Influenza, Human | D007251 | Pneumonia | D011014 |
| Asthma | D001249 | Pulmonary Disease, Chronic Ol | D029424 |
| Immune System Diseases | D007154 | Pulmonary Heart Disease | D011660 |
| Alzheimer Disease | D000544 | Schizophrenia | D012559 |
| Bipolar Disorder | D001714 | Schizophrenia | D012559 |
| Huntington Disease | D006816 | Schizophrenia | D012559 |
| Dengue Hemorrhagic Fever | D019595 | Testicular Neoplasms | D013736 |

| | | | |
|---|---|---|---|
| Diabetes, Gestational | D016640 | Varicocele | D014646 |
| Adrenal Cortex Neoplasms | D000306 | Vasculitis | D014657 |
